# Scoring protein-ligand binding structures through learning atomic graphs with inter-molecular adjacency

**DOI:** 10.1371/journal.pcbi.1013074

**Published:** 2025-05-09

**Authors:** Debby D. Wang, Yuting Huang

**Affiliations:** School of Science and Technology, Hong Kong Metropolitan University, Ho Man Tin, Hong Kong; Shiraz University, IRAN, ISLAMIC REPUBLIC OF

## Abstract

With a burgeoning number of artificial intelligence (AI) applications in various fields, biomolecular science has also given a big welcome to advanced AI techniques in recent years. In this broad field, scoring a protein-ligand binding structure to output the binding strength is a crucial problem that heavily relates to computational drug discovery. Aiming at this problem, we have proposed an efficient scoring framework using deep learning techniques. This framework describes a binding structure by a high-resolution atomic graph, places a focus on the inter-molecular interactions and learns the graph in a rational way. For a protein-ligand binding complex, the generated atomic graph reserves key information of the atoms (as graph nodes), and focuses on inter-molecular interactions (as graph edges) that can be identified by introducing multiple distance ranges to the atom pairs within the binding area. To provide more confidence in the predicted binding strengths, we have interpreted the deep learning model from the model level and in a post-hoc analysis. The proposed learning framework has been demonstrated to have competitive performance in scoring and screening tasks, which will prospectively promote the development of related fields further.

## Author summary

The binding between a small compound (ligand) and a protein plays a crucial role in many biological processes, such as signal transduction and immunoreaction. Particularly, a small-molecule drug can bind to a target protein to modulate its signaling pathways and suppress the progression of the associated disease. Apparently, the binding strength is a key indicator for evaluating how well such small-molecule drugs work, therefore becoming a core topic in computational drug discovery. Nowadays, the binding structure of a ligand and its target protein can be resolved experimentally or modeled computationally, while the accurate scoring of such a binding structure (predicting the binding strength) still remains a challenge. An effort has been put into the development of benchmark databases that provide a variety of protein-ligand binding structures and their experimentally resolved binding strengths, leading to increasing deep learning applications in this field. In this study, we represent a protein-ligand binding structure as a graph, with the atoms as nodes and the inter-molecular interactions as edges. A light but efficient deep learning architecture has been adopted for learning such graphs and outputting the binding strengths. Validated by our experiments, the model performs well in both scoring and screening tasks.

## Introduction

‘AI for science has attracted considerate attention in the past decade. Quite a number of powerful mathematical algorithms have been developed in this field, to rise to the challenging tasks in dermatology [1], precision medicine [2], molecular science [3] and drug discovery [4].

**Fig 1:**
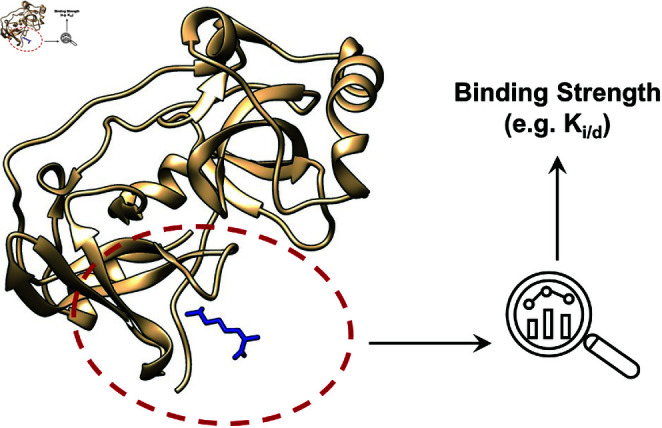
Scoring problem. Scoring a protein-ligand complex structure to exhibit the binding strength.

As a crucial problem in computer-aided drug discovery (CADD), scoring a protein-ligand complex structure to exhibit its binding strength (Fig 1) always seeks for breakthroughs in AI developments. Such binding strength, as a key indicator of the efficacy of a drug that attaches to its target protein, can mostly be attributed to various non-covalent interactions (e.g. hydrogen bonds, hydrophobic contacts and π-stacking). Earlier AI-based scoring works leveraged traditional machine-learning algorithms (e.g. random forests) to parse a feature vector, which describes the interactions in a protein-ligand complex structure, and mapped the vector to the binding strength [5–10]. It lasts until the emergence of deep learning, which reached its scientific milestones by the launch of *AlphaFold* (for near-perfect protein-fold predictions) [11] and the *GPT*-series (strong human-like chatbots) [12].

When first introduced to the works of scoring molecular binding strength, deep learning was primarily utilized in the manner of convolutional neural networks (CNNs) [13–18]. Accordingly, molecular lattices or grids, with each cell characterized by a collection of atomic properties (e.g. physico-chemical or pharmacophoric), are the *de facto* feature representations of a protein-ligand complex. The KDEEP model adopts a molecular lattice representation with a size of 24Å×24Å×24Å and a set of eight atomic properties (pharmacophoric) for delineating a complex structure, and feeds the lattice into a 3D-CNN for binding strength prediction [13]. The Pafnucy model compresses 19 atomic properties (both physico-chemical and pharmacophoric) of a complex structure into a molecular lattice (20Å×20Å×20Å), and employs a simple 3D-CNN architecture for learning the lattice [14]. Rezaei *et al*. developed a light-weight 3D-CNN model for scoring, based on 32Å×32Å×32Å molecular lattices that concern 24 atomic features (11 Arpeggio atom types and the excluded volume for both protein and ligand) [15]. Although has opened a new venue for scoring works, deep lattice learning often lacks rotational invariance in data and is therefore resource-intensive after data augmentation [13, 14].

More recently, molecular graph learning has become a prevalent technique for the scoring works. In this context, a protein-ligand complex structure is commonly represented as a 2D atomic graph, which is then decoded by graph neural networks (GNNs) [19–23]. GraphBAR adopts a molecular graph with distance-dependent edges to characterize the binding-site atoms in a complex structure, and employs a spectral graph convolutional network (GCN) to map the graphs to the binding strengths [20]. Shen *et al*. considered the covalent connections for atoms in the binding area, and leveraged a cascade GCN (with two concatenated modules) for graph learning and binding strength prediction [21]. GraphscoreDTA represents a complex by a fusion of graphs (a 1D amino-acid graph for protein, an atomic graph for ligand, and a hybrid graph for the binding pocket), and predicts the binding strength using a GNN with a bitransport information mechanism and Vina distance terms [23]. Zhang *et al*. utilized a similar graph representation and developed a multi-objective GNN model for binding-strength scoring [22]. These pioneer works have shed light on modern scoring works. Nevertheless, there is still much room for improvement in developing target-oriented graph representation, achieving high screening power and making the model more transparent. Accordingly, we are dedicated to the design of efficient scoring models, with informative molecular graphs, descent screening power and reasonable interpretability, in this work.

## Materials and methods

### Atomic-level molecular graphs

A molecular graph can be represented as 𝒢=(𝐕,𝐄), where **V** indicates nodes $\left\{nd_{1}^{0},\ldots ,nd_{\left|V\right|}^{0}\right\}$ and **E** stands for edges connecting those nodes $\left\{eg_{1}^{0},\ldots ,eg_{\left|E\right|}^{0}\right\}$.

To capture sufficient information in a molecule, treating its atoms as graph nodes is a well-acknowledged strategy. Each node or atom is then characterized by a series of physico-chemical or pharmacophoric properties, leading to a feature matrix ${𝐅}^{0}\in {\mathbb{R}}^{|𝐕|\times m}$ of all the nodes in the graph (*m* is the number of properties). As molecules like proteins are very large in terms of atoms, retaining all the atoms is a heavy burden to the computations and therefore task-oriented cropping is frequently performed. For scoring a protein-ligand binding structure, the atoms in the binding area is often of interest. This results in a smaller feature matrix $𝐅\in {\mathbb{R}}^{n\times m}$, where *n* is the number of nodes in the binding area (${𝐕}^{ba}=\{nd_{1},\ldots ,nd_{n}\}$).

**Fig 2:**
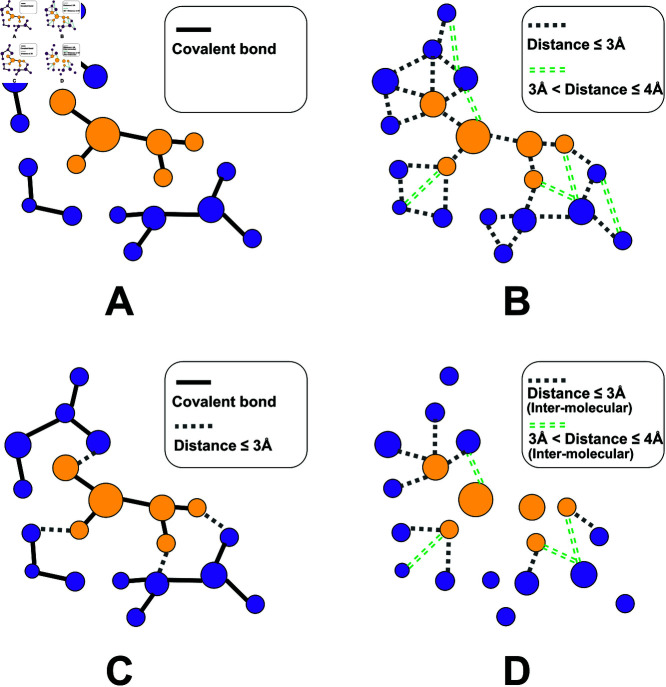
Different definitions of atomic adjacency in a molecular graph. **A**. Covalent adjacency. **B**. Distance-dependent contacts. **C**. A combination of covalent adjacency and distance-dependent contacts. **D**. Inter-molecular contacts through distance thresholding.

Generally, a graph in deep learning works shows the connections between nodes by an adjacency matrix **A**, where *A*_*ij*_ indicates an edge between the *i*-th and *j*-th nodes. However, designing task-specific graph edges, especially for tasks involving molecules, is often challenging. The covalent bonds, contacts defined through distance thresholding, or a combination of them have been regarded as edges in different works [20, 21, 24]. Considering the atoms in the binding area of a protein-ligand complex, Fig 2A shows the covalent adjacency among those atoms. Interactions or contacts between a pair of atoms (*nd*_*i*_ and *nd*_*j*_) can also be defined by the range where the atomic distance (*d*_*ij*_) resides, leading to multi-level distance-dependent adjacencies among atoms. Fig 2B displays two types of atomic contacts ($d_{ij}\leq 3Å$ and $3Å<d_{ij}\leq 4Å$). In Fig 2C, a hybrid type of adjacencies (covalent bond and distance-dependent contacts) is considered. *For scoring tasks, these adjacency definitions either emphasize the covalent bonds, or mix the inter- and intra-molecular interactions, resulting in the loss of focus on the inter-molecular interactions.* Nevertheless, these inter-molecular interactions play a pivotal role in determining the binding strength between a ligand and its target protein. Accordingly, we focus on the inter-molecular contacts in this work, and define multi-level atomic adjacencies by one-hot encoding of those contacts belonging to different distance ranges (Fig 2D). Such adjacencies can be stored in an adjacency tensor **A**, where each slice ${𝐀}_{::k}$ shows all pairs of nodes having distances in range $\delta_{k}$, as follows.

$$A_{ijk}=\left\{ \begin{array}{cc} 1 & {\mathrm{nd}}_{i}\text{ and }{\mathrm{nd}}_{j}\text{ is a protein-ligand atom pair}\\ & \&\;d_{ij}\in \delta_{k}\\ 0 & otherwise \end{array}\right.$$ (1)

Algorithm 1 shows the procedure for generating such an adjacency tensor for a protein-ligand binding area.

**Algorithm 1** Generating an Inter-molecular Adjacency Tensor

**Input:** Coordinates $Coord_{1},\ldots ,Coord_{n}$ for the atoms in the binding area ($nd_{1},\ldots ,nd_{n}$), a list of distance ranges $\delta_{1},\ldots ,\delta_{K}$

**Output:** An inter-molecular adjacency tensor **A**

Initialize 𝐀=0 ($\in {\mathbb{R}}^{n\times n\times K}$).

Calculate the distance matrix $𝐃\in {\mathbb{R}}^{n\times n}$ based on $Coord_{1},\ldots ,Coord_{n}$.

**for** *k* = 1 **to** *K* **do**

${𝐀}_{::k}={𝐈}_{𝐃\in \delta_{k}}$         ⊳ ${𝐈}_{𝐃\in \delta_{k}}$ is an indicator function

**for** *i* = 1 **to** *n* **do**

**for** *j* = *i* + 1 **to** *n* **do**

**if** $A_{ijk}\neq 0$ and *nd*_*i*_-*nd*_*j*_ is not a protein-ligand atom pair **then**

$A_{ijk}=A_{jik}=0$         ⊳ Turn off intra-molecular interactions

**end if**

**end for**

**end for**

**end for**

### Graph-based deep learning

Given a graph with a node-feature matrix **F** and an adjacency matrix **A**, message-passing mechanisms are frequently adopted for learning such a graph [25]. These mechanisms, as shown in Eq 2, update nodes features iteratively in a local context.

$${𝐟}_{i}^{l+1}=\alpha_{l}({𝐟}_{i}^{l},{⊕}_{j\in 𝒩(i)}{𝐦}_{j\to i}^{l})$$ (2)

Here, ${𝐟}_{i}^{l}$ is the features describing the *i*-th node in the *l*-th layer, 𝒩(i) indicates the neighborhood of the *i*-th node (based on the adjacency matrix), ${𝐦}_{j\to i}^{l}$ is the message passed from the *j*-th node to the *i*-th node in the *l*-th layer, ⊕ denotes a permutation-invariant function (e.g. average), and $\alpha_{l}$ is an update function such as a neural network.

Although a wide variety of graph neural networks (GNNs) have been developed, properly learning molecular graphs still remains a challenge. The *ChebNet* [26], leveraging spectral graph convolutions, is among the well-acknowledged GNNs. It has an efficient form for updating node features in each iteration, as follows.

$${𝐅}^{l+1}=\sigma_{l}({\tilde{𝐃}}^{-\frac{1}{2}}\tilde{𝐀}{\tilde{𝐃}}^{-\frac{1}{2}}{𝐅}^{l}{𝐖}^{l})$$ (3)

Here, ${𝐅}^{l}$ is the feature matrix for all the nodes in the *l*-th layer, $\sigma_{l}$ is an activation function, ${𝐖}^{l}$ is the weight matrix, and ${\tilde{𝐃}}^{-\frac{1}{2}}\tilde{𝐀}{\tilde{𝐃}}^{-\frac{1}{2}}$ is a normalized adjacency matrix with self-adjacencies ($\tilde{𝐀}=𝐀\;+\;{𝐈}_{n}$ and ${\tilde{D}}_{ii}=\sum_{j}{\tilde{A}}_{ij}$). Such graph-learning operations can be stacked into *L* layers. From the message-passing perspective, this mechanism can be regarded as a simple average of the normalized information collected from the neighborhood of a node.

$${𝐟}_{i}^{l+1}=\alpha_{l}(\frac{1}{\tilde{D_{ii}}}{𝐟}_{i}^{l}{𝐖}^{l}+\sum_{j\in 𝒩(i)}\frac{{\tilde{A}}_{ij}}{\sqrt{\tilde{D_{ii}}\tilde{D_{jj}}}}{𝐟}_{j}^{l}{𝐖}^{l})$$ (4)

Previously, *ChebNet* has been questioned about its capability for capturing long-range dependence among the nodes in a graph. *However, scoring protein-ligand binding strength is a work that largely concerns local contexts (e.g. a key hydrogen bond or an important interaction), making the ChebNet mechanism fit well in this task.* When focusing on only the inter-molecular interactions (Fig 2D), we update the features once (Eq 3) to learn the neighborhoods of binding-site atoms in this work. Higher-order graph convolutions, which will involve intra-molecular interactions and be computationally expensive, are not considered. This strategy places an absolute focus on the inter-molecular interactions (crucial to scoring works) and is of high efficiency. Since *L* = 1 in this scenario, the layer notation *l* will be omitted for simplicity in what follows.

Instead of using a single adjacency matrix **A**, an adjacency tensor that covers different adjacency (interaction) types is of necessity in a scoring task. As inter-molecular interactions are mostly non-covalent (atomic distance $d_{ij}>2Å$), multiple distance ranges starting from 2Å can be nominated to construct the inter-molecular adjacency tensor. Fig 2D exhibits a two-slice adjacency tensor **A** as follows.

$$A_{ij1}=\left\{ \begin{array}{cc} 1 & {\mathrm{nd}}_{i}\text{ and }{\mathrm{nd}}_{j}\text{ is a protein-ligand atom pair}\\ & \&\;2Å<d_{ij}\leq 3Å\\ 0 & otherwise \end{array}\right.\;A_{ij2}=\left\{ \begin{array}{cc} 1 & {\mathrm{nd}}_{i}\text{ and }{\mathrm{nd}}_{j}\text{ is a protein-ligand atom pair}\\ & \&\;3Å<d_{ij}\leq 4Å\\ 0 & otherwise \end{array}\right.$$ (5)

Here we adopt such a tensor because 4Å has been verified to be a distance threshold for capturing sufficient inter-molecular interactions in a binding complex [20].

Targeting at each type of inter-molecular contacts (k=1,2), the graph nodes can be learned using the message-passing mechanism in *ChebNet*, as

$${𝐅}^{k}=\sigma ({\tilde{𝐃}}_{k}^{-\frac{1}{2}}{\tilde{𝐂}}_{k}{\tilde{𝐃}}_{k}^{-\frac{1}{2}}𝐅{𝐖}_{k})$$ (6)

where ${\tilde{𝐂}}_{k}={𝐀}_{::k}+{𝐈}_{n}$, ${\tilde{𝐃}}_{k}$ is a diagonal matrix showing the degree of each node in ${\tilde{𝐂}}_{k}$, and all the other notations follow Eq 3.

After collecting the messages from direct neighbors of graph nodes, we gather the information into the graph level for the scoring purpose. Such an aggregation function is permutation-invariant and similar to that in Eq 2. A simple summation in the following equation serves as an example.

$${𝐡}_{k}=\sum_{i\in {𝐕}^{ba}}{𝐟}_{i}^{k}$$ (7)

The features for different inter-molecular interactions (k=1,2) are then concatenated before being fed into dense layers for final graph-level predictions.

$$𝐡={\|}_{k}{𝐡}_{k}$$ (8)

Here, ‖ indicates an concatenation of features and **h** stands for the hidden features describing the whole binding-site graph.

**Fig 3:**
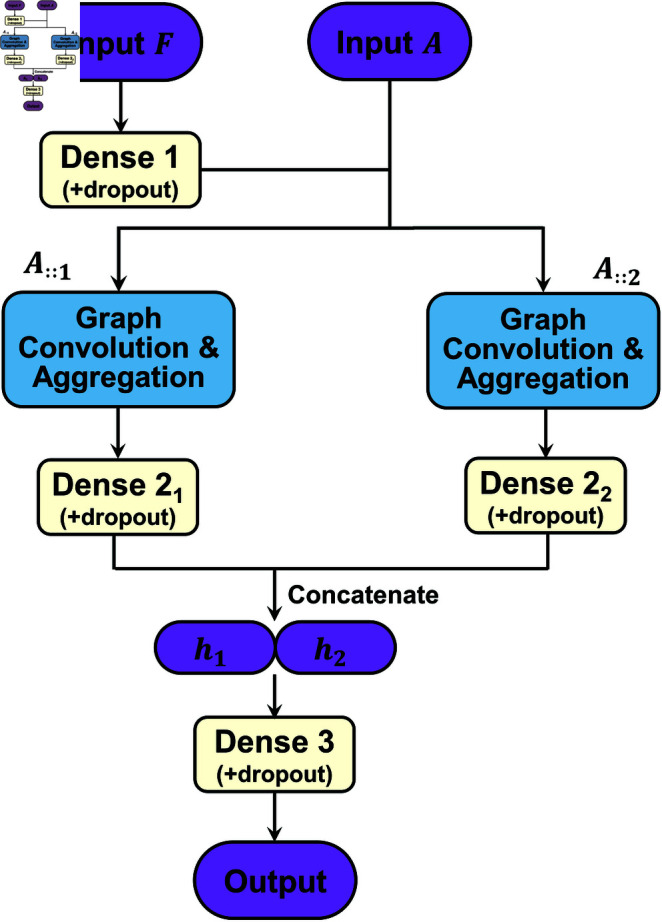
A light graph-learning architecture adopted in this work. The node feature matrix **F** and inter-molecular adjacency tensor **A** of a protein-ligand complex are the inputs, and the binding strength is the output. Main components of this architecture include graph convolution layers, node aggregation layers, dense (fully-connected) layers and dropout layers.

Referring to a well-established architecture (*GraphBAR* [20]), we developed a light graph-learning architecture that focuses only on inter-molecular interactions and learns the interactions through direct atomic neighborhoods (Fig 3).

## Experiment and results

### Scoring performance of models

The aforementioned framework scores the binding strengths of protein-ligand complex structures through learning Atomic Graphs with Inter-Molecular Adjacency (AGIMA-based scoring, abbreviated as *AGIMA-Score*). In an *AGIMA-Score* model, the binding area of a complex structure is treated as a graph, represented by a node-feature matrix $𝐅\in {\mathbb{R}}^{n\times m}$ and an adjacency tensor $𝐀\in {\mathbb{R}}^{n\times n\times K}$. Here the binding area is recognized as all the ligand atoms and the protein atoms within 4Å-distance of any ligand atom, referring to Son’s work [20]. Three sets of node features, referring to *Pafnucy* (${𝐅}^{18}$, *m* = 18) [14], *KDEEP* (${𝐅}^{8}$, *m* = 8) [13] and *GraphBAR* [20] (${𝐅}^{13}$, *m* = 13) respectively, were adopted to construct **F** (Table 1). ${𝐅}^{18}$ includes *generic physico-chemical* properties (e.g. atom types and partial charge) and *pharmacophoric* properties (e.g. aromaticity and hydrogen-bond membership) of atoms. ${𝐅}^{13}$ is a subset of ${𝐅}^{18}$ that excludes *pharmacophoric* properties. ${𝐅}^{8}$ focuses on *pharmacophoric* properties, with atomic charge and excluded volume considered.

**Table 1:** Node-feature sets for building molecular graphs. Three feature sets, with 18 features (from *Pafnucy*), 8 features (from *KDEEP*) and 13 features (from *GraphBAR*) respectively, were considered in this study. The names and data types of these features are listed.

Feature set	Components	Data type
${𝐅}^{18}$	$f_{1}\sim f_{9}$: one-hot encoding of atom types	
	(Boron, Carbon, Nitrogen, Oxygen, Phosphorus, Sulphur, Selenium, Halogen atom and Metal atom)	
	*f*_10_: hybridization type	$f_{1}\sim f_{9}$: binary
	*f*_11_: number of heavy-atom neighbors	$f_{10}\sim f_{12}$: integer
	*f*_12_: number of hetero-atom neighbors	$f_{13}\sim f_{17}$: binary
	$f_{13}\sim f_{17}$: pharmacophoric features (hydrophobicity, aromaticity, hydrogen-bond acceptor, hydrogen-bond donor and ring membership)	*f*_18_: float
	*f*_18_: partial charge	
${𝐅}^{8}$	$f_{1}\sim f_{5}$: pharmacophoric features (hydrophobicity, aromaticity, hydrogen-bond acceptor, hydrogen-bond donor and metallicity)	$f_{1}\sim f_{5}$: binary
	*f*_6_: positive charge	$f_{6}\sim f_{8}$: float
	*f*_7_: negative charge	
	*f*_8_: excluded volume	
${𝐅}^{13}$	$f_{1}\sim f_{13}$: features in ${𝐅}^{18}$ except the pharmacophoric features ($f_{13}\sim f_{17}$)	$f_{1}\sim f_{9}$: binary $f_{10}\sim f_{12}$: integer
		*f*_13_: float

When constructing the inter-molecular adjacency tensor **A** (Fig 2D), two distance ranges were selected for capturing multi-level protein-ligand interactions, as follows. As a pair of atoms having a distance <2Å are mostly connected by a covalent bond, we paid more attention to the atom pairs being >2Å apart for characterizing the inter-molecular interactions (non-covalent). Meanwhile, the binding area is recognized according to a pairwise atomic distance of <4Å, we employed the two distance ranges, (2Å,3Å) and (3Å,4Å), in Eq 5 to build the adjacency tensor ($𝐀\in {\mathbb{R}}^{n\times n\times 2}$) in this work. Combining the three node-feature matrices (${𝐅}^{18}$, ${𝐅}^{8}$ and ${𝐅}^{13}$) and the adjacency tensor ( **A**) in the generation of molecular graphs, we constructed three *AGIMA-Score* models (*AGIMA-Score*^18^, *AGIMA-Score*^8^ and *AGIMA-Score*^13^) based on the graph-learning architecture in Fig 3. To investigate whether a single distance range of (2Å,4Å) can cover sufficient adjacency information, we built a single-matrix adjacency tensor (${𝐀}_{SAM}\in {\mathbb{R}}^{n\times n\times 1}$) to pair up with the three node-feature matrices for each protein-ligand complex. This led to the construction of three new models (*AGIMA-Score*${\;}_{SAM}^{18}$, *AGIMA-Score*${\;}_{SAM}^{8}$ and *AGIMA-Score*${\;}_{SAM}^{13}$) for comparison purpose. In addition, the non-redundant features (${𝐅}^{21}$) from ${𝐅}^{18}\cup {𝐅}^{8}\cup {𝐅}^{13}$ were collected and combined with the adjacency tensor **A** to build the *AGIMA-Score*^21^ model. The dense layers each have a dimension of 128, and the number of epochs and batch size were tuned when constructing these models.

In order to evaluate the performance of these models comprehensively, several broadly-discussed, deep-learning scoring models were implemented as competing models. These include the Atom Convolutional Neural Network (*ACNN*) [27], *OnionNet* [18], *KDEEP* [13] and *GraphBAR* [20]. For *ACNN*, parameters including pooling filters, number of epochs and batch size were tuned in our work to reach the best model. Number of epochs and batch size were tuned for *OnionNet* and *KDEEP* (no data augmentation). As two similar graph-learning approaches, *GraphBAR* considers both intra- and inter-molecular contacts (Fig 2B) while *AGIMA-Score* focuses on only the inter-molecular contacts (Fig 2D) in the construction of molecular graphs. To make a fair comparison with *AGIMA-Score* models, we constructed two *GraphBAR* models, *GraphBAR*_2*AM*_ and *GraphBAR*_3*AM*_, based on the architecture in Fig 3. *GraphBAR*_2*AM*_ takes into account the intra-/inter-molecular contacts within (0,2Å) and those within (2Å,4Å) when building the adjacency tensor, which corresponds to the *AGIMA-Score*_*SAM*_ models (considering inter-molecular contacts in (2Å,4Å)). *GraphBAR*_3*AM*_ adopts three distance ranges ((0,2Å), (2Å,3Å) and (3Å,4Å)) to collect the intra-/inter-molecular adjacencies, corresponding to the *AGIMA-Score* models (considering inter-molecular contacts in (2Å,3Å) and (3Å,4Å)). The number of epochs and batch size were treated as tuning parameters for the two *GraphBAR* models.

**Fig 4:**
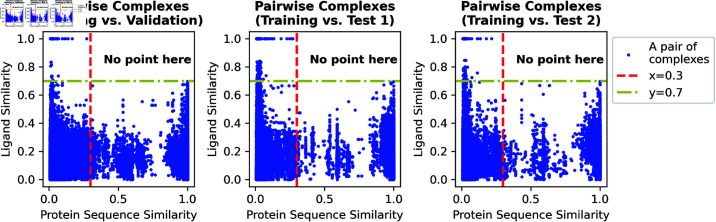
Similarity test for each pair of complexes (Training vs. Validation, Training vs. Test1, and Training vs. Test2). The horizontal axis stands for the similarity between the two protein sequences involved in a complex pair, and the vertical axis indicates the similarity between the two involved ligands. The red dotted line means a sequence similarity of 0.3 and the yellow line shows a ligand similarity of 0.7.

The *AGIMA-Score* and competing models were constructed based on the benchmark *PDBbind* database (https://www.pdbbind-plus.org.cn/). The *Refined Set* and *Core Set* in this database were employed for training and parameter tuning (validation). Each sample in these two sets is a protein-ligand complex structure (determined mostly by X-ray crystallography or NMR spectroscopy) with the experimentally resolved binding strength (−*logK*_*d*/*i*_). These structural and binding-strength data are of high quality as they have gone through rigorous filtering processes [28, 29]. To avoid potential train-validation contamination, each pair of complexes, one from the validation set and the other from the training set, needs to pass a similarity test. This test guarantees that the similarity of two protein sequences is below 0.3 or the similarity of two ligands is below 0.7, in each pair of complexes. Protein sequence similarities were generated using the *crossSetSim* function from the *protr* R library, with the default BLOSUM62 substitution matrix. Ligand similarities were calculated using the *cmp.similarity* function from the *ChemmineR* library, based on SMILES-transformed descriptors. The complexes against this rule were removed from the *Validation* set. Two sets from *CSAR* [30] were regarded as the final test sets, named *Test1* and *Test2*, in case of the over-optimistic results yielded from using the same-source data sets. The aforementioned similarity test was also performed on each test set vs. training set to prevent from potential train-test contamination. After this cleaning, the similarity statistics for pairwise complexes, with one complex from the training set and the other from the Validation, Test1, or Test2 set, are presented in Fig 4. Furthermore, the same protocol was adopted to ensure that there was no contamination among the *Validation*, *Test1* and *Test2* sets. Finally, the filtered *Training*, *Validation*, *Test1* and *Test2* sets consist of 5007, 195, 116 and 102 complex structures respectively. The lists of complexes for these sets can be found in *Zenodo* (https://zenodo.org/records/15023336).

The performance of each model was evaluated according to (1) the Pearson’s Correlation (PC) between the experimental and predicted binding strengths of the complex structures and (2) the room-mean-square-error (RMSE) concerning those binding strengths. The evaluation results are now listed in Table 2.

**Table 2:** Scoring Performance Comparison. The models were trained on *PDBbind Refined Set* (version *V2020*) with parameters tuned via the *Core Set* (version *V2020*), and tested on two sets from the *CSAR* source. State-of-the-art deep learning models (*ACNN*, *OnionNet*, *KDEEP* and *GraphBAR*) for scoring the protein-ligand complexes were realized, to comprehensively evaluate the proposed *AGIMA-Score* models. For *GraphBAR*, different graph adjacency schemes (2 or 3 adjacency matrices) were adopted for model construction. For *AGIMA-Score*, different node features (separately referring to *Pafnucy*, *KDEEP* and *GraphBAR*) and adjacency schemes (2 adjacency matrices or single adjacency matrix) were considered for model investigation. By default, 2 adjacency matrices (generated by intermolecular atomic contacts within (2Å,3Å) and those within (3Å,4Å)) were adopted in the graph learning by *AGIMA-Score*. Best performance in terms of PC and RMSE were underlined for the state-of-the-art methods and the proposed *AGIMA-Score* models.

Model	Training		Validation		Test1		Test2	
	PC	RMSE	PC	RMSE	PC	RMSE	PC	RMSE
*ACNN*	0.5225	1.7040	0.6586	1.7433	0.6079	1.8017	0.6064	1.7243
*OnionNet*	0.8745	1.0405	0.8663	1.3036	0.6547	1.7742	**0.6543**	1.6682
*KDEEP*	0.9909	0.2623	0.8310	1.2728	0.5339	1.9973	0.5963	1.7375
*GraphBAR*_2*AM*_	0.5906	1.5783	0.6736	1.7748	**0.7192**	1.6557	0.6540	**1.6557**
*GraphBAR*_3*AM*_	0.6184	1.5717	0.6922	1.6739	0.7178	**1.6459**	0.6450	1.6916
*AGIMA-Score* ^18*^	0.6707	1.4638	0.7262	1.6949	0.7339	**1.5806**	**0.6698**	**1.6279**
*AGIMA-Score* ${\;}_{SAM}^{18*}$	0.6540	1.4732	0.6978	1.6961	0.7154	1.6211	0.6042	1.7027
*AGIMA-Score* ^8 + ^	0.5533	1.6260	0.6976	1.7594	**0.7414**	1.6740	0.6466	1.6989
*AGIMA-Score* ${\;}_{SAM}^{8+}$	0.5466	1.6313	0.7018	1.7351	0.7123	1.6860	0.6686	1.6509
*AGIMA-Score* ^13@^	0.6351	1.5396	0.7096	1.6912	0.7156	1.7102	0.6534	1.7456
*AGIMA-Score* ${\;}_{SAM}^{13@}$	0.6023	1.5611	0.6634	1.7776	0.7177	1.6780	0.6398	1.7081
*AGIMA-Score* ${\;}^{21\#}$	0.6319	1.5180	0.6855	1.7478	0.7413	1.6327	0.6677	1.6549

2*AM* - 2 adjacency matrices (generated by atomic contacts within (0,2Å) and those within (2Å,4Å)) were used in the graph learning by *GraphBAR*.

3*AM* - 3 adjacency matrices (generated by atomic contacts within (0,2Å), contacts within (2Å,3Å) and those within (3Å,4Å)) were used in the graph learning by *GraphBAR*.

^*^ Using 18 features for characterizing graph nodes (referring to *Pafnucy*).

^ + ^ Using 8 features for characterizing graph nodes (referring to *KDEEP*).

^@^ Using 13 features for characterizing graph nodes (referring to *GrahBAR*).

${\;}^{\#}$ Using 21 features for characterizing graph nodes (non-redundant features of those from *Pafnucy*, *KDEEP* and *GrahBAR*).

*SAM* - Single adjacency matrix (generated by intermolecular atomic contacts within (2Å,4Å)) was adopted in the graph learning by *AGIMA-Score*.

An *ACNN* model is underfitted easily while a *KDEEP* model is often overfitted. Among the earlier models (*ACNN*, *OnionNet*, *KDEEP* and *GraphBAR*), *GraphBAR* outperforms the others in terms of *Test1*-PC, *Test1*-RMSE and *Test2*-RMSE, while *OnionNet* reaches the best PC on *Test2* set. Compared to these earlier models, the *AGIMA-Score* models perform well in average. *AGIMA-Score*^18^ achieves the overall best performance because of the lowest *Test1*-RMSE, highest *Test2*-PC and lowest *Test2*-RMSE. *AGIMA-Score*^8^ attains the best performance with respect to *Test1*-PC. Although using a single adjacency matrix (*AGIMA-Score*_*SAM*_) eases the computations in graph learning, it often results in an underperformance in terms of PC and RMSE (compared to *AGIMA-Score*). It shows that using two adjacency matrices captures more information about the connections among atoms, such as the strong and weak hydrogen bonds with donor-acceptor distances of 2.2~2.5Å and 3.2~4.0Å separately [31]. The outperformance of *AGIMA-Score* over *GraphBAR* demonstrates the efficacy of learning the molecular graphs with inter-molecular adjacencies, rather than mixed intra- and inter-molecular adjacencies, in a scoring task. Overall, these results reveal the strong competitiveness of *AGIMA-Score* models in scoring the binding strength of a protein-ligand complex. A Docker container with the trained *AGIMA-Score*^18^ model pre-installed is available for access on *Zenodo* (https://zenodo.org/records/15023336).

### Screening performance of models

**Fig 5:**
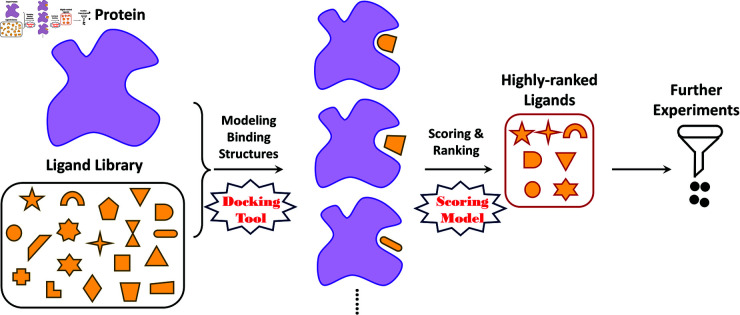
A virtual screening task. The task starts from a target protein and a big library of ligands, followed by the modeling of each protein-ligand binding structure (docking tool) and the scoring of the binding structures (scoring model). The highly-ranked ligands (according to the predicted scores) will be regarded as potential binders for further biochemical experiments.

The scoring performance shows the capability of ranking a list of binding complexes or predicting the accurate binding strengths. Beyond that, the screening power is another indicator of interest for further evaluating the prediction models. As a more practical task, virtual screening aims to discover the potential binders for a target protein, in order to mitigate the burden of downstream biochemical experiments (Fig 5). Such a target protein often plays a key role in regulating the progression of some diseases, exemplified by the epidermal growth factor receptor (EGFR) protein that mediates the growth of non-small-cell lung cancer (NSCLC). Modeling the binding structure of each protein-ligand pair (*docking*) and scoring the binding strength based on this structure (*scoring*) are the two primary subtasks in virtual screening. Current state-of-the-art docking tools (e.g. *AUTODOCK* [32] and *Glide*[33]) can provide near-experimental binding structure for a pair of protein and ligand, while accurately scoring such a binding structure has long been a challenge. This work aims mainly at the scoring phase. Once we have the scored binding structures according to a model, *whether the true binders for the target protein can be highly ranked* is a main indicator of the screening capability of this model. *A model with both high scoring and high screening power is always the pursuit of the CADD community.*

To evaluate the screening power of *AGIMA-Score* models and their competitors, we selected a comparably large set from the *DUD-E* source (https://dude.docking.org). This set concerns the aforementioned EGFR and its potential ligands. A total of 36,273 ligands have been included in this set, with 832 actives (binders) and 35,441 decoys (non-binders) for the EGFR protein. Noteworthily, the models discussed above accept protein-ligand binding structures as inputs, but this *EGFR* set only contains the structures of the monomers (EGFR protein and ligands). Accordingly, we paired up the protein with each ligand into a binding structure by the well-acknowledged *AUTODOCK Vina* docking tool, before feeding the structures into the models. The best binding pose was retained for each protein-ligand pair, based on the default setting in *AUTODOCK Vina* and a reference structure (PDB:2RGP). The generated 36,273 EGFR-ligand binding structures were then fed into each model (*ACNN*, *OnionNet*, *KDEEP*, *GraphBAR*_2*AM*_, *GraphBAR*_3*AM*_, *AGIMA-Score*^18^, *AGIMA-Score*^8^ and *AGIMA-Score*^13^) to predict the binding strengths.

Enrichment factor (EF) is a widely-used index for evaluating the screening performance of a scoring model. It is defined as $EF^{X}=\frac{Y\%}{X\%}$, where Y% is the percentage of actives in the top X% ranked ligands. Meanwhile, the total decoy-to-active ratio ($r_{DTA}^{max}$) for this set is $\frac{35,441}{832}\approx 43$, indicating a high imbalance between actives and decoys. To provide a more comprehensive evaluation, we composed a series of secondary sets according to varying *r*_*DTA*_ values and assessed the corresponding EFs for each model on these sets. Aiming at a specific model **MDL**, an *r*_*DTA*_ ($r_{DTA}=2,\ldots ,r_{DTA}^{max}$) and an *X* value, this procedure is described as follows.

- Keep all the 832 actives and randomly select $832\times r_{DTA}$ decoys to constitute a set of size $832\times (1+r_{DTA})$.
- According to **MDL**, score and rank all the EGFR-ligand complexes in each of the set generated above, and calculate $EF_{r_{DTA}}^{X}$.
- Repeat the process for 10 times to derive the average EF ($\overset{―}{EF_{r_{DTA}}^{X}}$).

**Fig 6:**
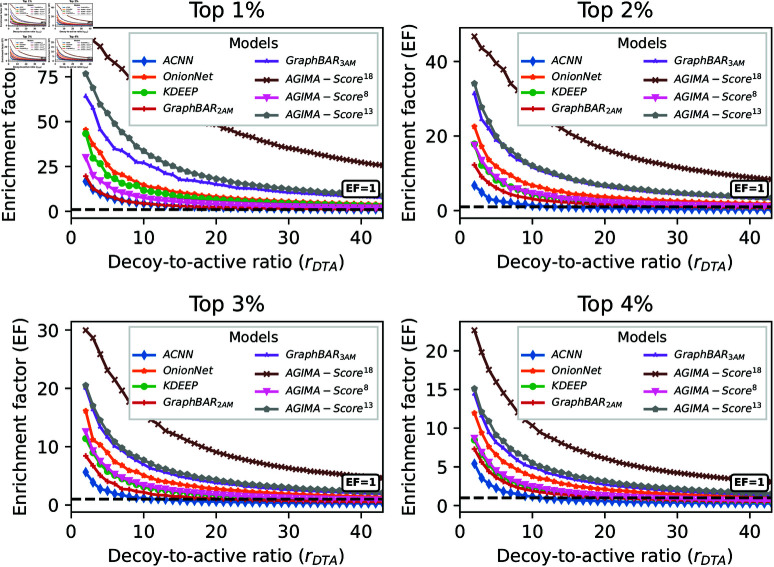
The screening performance of each model on the EGFR set, with the top 1~4% ranked ligands considered. In each scenario, the enrichment factors (EFs) regarding various decoy-to-active ratios (*r*_*DTA*_) were calculated for each model, and plotted in a line. The black dashed lines indicate EF = 1.

The top 1~4% (X=1~4) ranked ligands were used for evaluating the screening performance of each model in Table 2, and the results are now exhibited in Fig 6.

**Fig 7:**
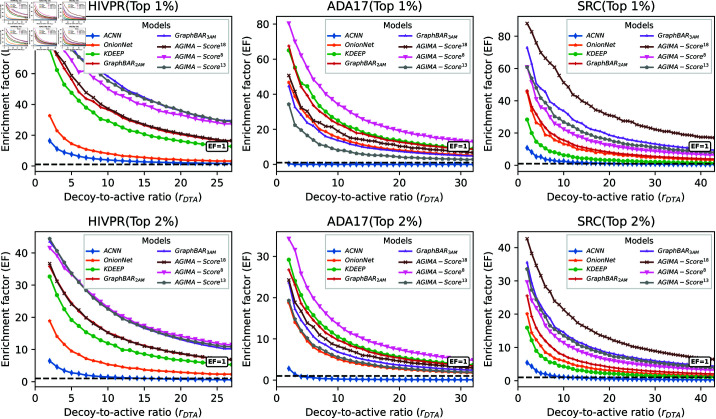
The screening performances of AGIMA-Score and competing models on the HIVPR, ADA17 and SRC sets, with the top 1~2% ranked ligands considered. In each scenario, the enrichment factors (EFs) regarding various decoy-to-active ratios (*r*_*DTA*_) were calculated for each model, and plotted in a line. The black dashed lines indicate EF = 1.

Here, EF goes worse as *r*_*DTA*_ or *X* goes larger for all the models. However, *AGIMA-Score*^18^ performs markedly better than the others. Encouragingly, *AGIMA-Score*^18^ even achieves an EF of 26 for the top 1% ranked ligands, when all the decoys are included in the assessment. Similar results for the top 5~8% ranked ligands are displayed in S1 Fig. Three more tasks, involving target proteins of HIV protease (HIVPR), ADAM17 Protease (ADA17) and tyrosine-protein kinase SRC (SRC), were considered. The *HIVPR* set covers 37,673 potential ligands (1,395 actives/36,278 decoys) for HIVPR protein. 30,956 (959 actives/29,997 decoys) and 35,790 (831 actives/34,959 decoys) ligands are included in the *ADA17* and *SRC* sets, respectively. The screening performance of the *AGIMA-Score* models and competitors on these three sets, in terms of the top 1~2% ranked ligands, are displayed in Fig 7. For *HIVPR* set, *AGIMA-Score*^13^ performs the best, with an EF of 29 for the top 1% ligands when involving all decoys. *AGIMA-Score*^8^ outperforms the others for the *ADA17* set and *AGIMA-Score*^18^ is the best performer for the *SRC* set, with EFs of 13 and 17 for the top 1% ligands (all ligands involved) respectively. The results for the top 3~4% ranked ligands are presented in S2 Fig. Such results promote the *AGIMA-Score* models further.

## Discussion on model interpretability

Interpretations of deep learning models can build confidence in their predictions, therefore attracting more and more attention in recent years. Here, we discuss the ways to interpret *AGIMA-Score* models from **model level** and **post-hoc analysis**.

### Model-level interpretation.

Due to the black-box nature of deep learning models, explaining the intrinsic structures of these models, which often concern millions or even more parameters, is quite difficult. In this regard, we focus mainly on the learning architecture (Fig 3) of *AGIMA-Score* models. This framework first transforms the original node features to an embedding space, and then considers multi-range, distance-dependent inter-molecular interactions ($2Å<d_{ij}\leq 3Å$ and $3Å<d_{ij}\leq 4Å$) between a pair of protein and ligand. They imply important local interaction patterns between the two binding molecules. After a further feature-embedding transformation (${𝐡}_{1}$ and ${𝐡}_{2}$), the framework gathers the information from those interaction patterns (by concatenation of ${𝐡}_{1}$ and ${𝐡}_{2}$) in the binding area. Then it maps the gathered information into the components of molecular binding strength or interaction energy using another hidden layer, leading to the final prediction of total binding strength. Hence, the framework can be partly explained in the perspective of molecular interaction energies.

### Post-hoc interpretation.

After a model is constructed, investigating the roles of different features in the decision-making process and monitoring the correlations between some hidden features and the outputs are well-acknowledged strategies for decoding the model in a post-hoc way. Specifically, we employed the *masking-based feature importance assessment* and *principal component analysis (PCA) of key feature embeddings* in our work.

***Masking-based feature importance assessment.*** To simplify the scenario, ascertaining the importance of each node feature in the decision-making process for a given *AGIMA-Score* model is the goal here. For such a model **MDL**, this assessment procedure is described as follows.

- Implement **MDL** on predicting the binding strengths of all the complexes in the validation set. Suppose the results are *PC*_0_ and *RMSE*_0_.
- Mask one node feature (*i*-th feature) at a time and re-implement **MDL** on the scoring task. Here, masking a feature means replacing the original features with 0s. As a result, a drop in PC ($\Delta PC_{i}$) and an increase in RMSE ($\Delta RMSE_{i}$) will be derived.
- Rank the node features in terms of PC drops (or RMSE increase), and those corresponding to a large PC drop (or RMSE increase) after being masked are more important in the decision-making process.

**Fig 8:**
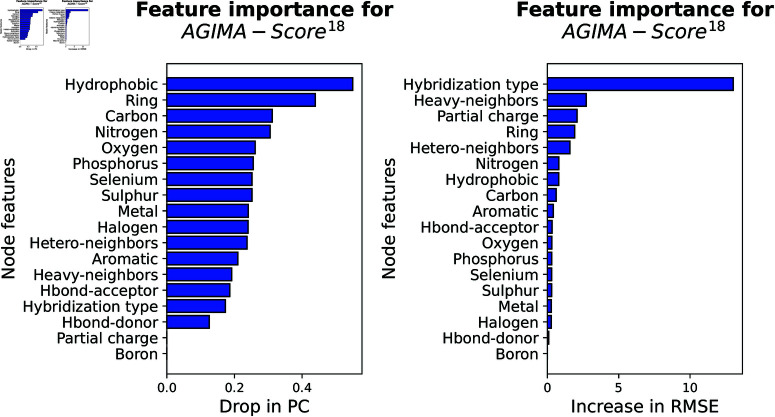
Importance assessment of the node features involved in the AGIMA-Score^18^ model. The result was revealed by the masking-based performance drop on the validation set (*PDBbind Core Set*).

**Fig 9:**
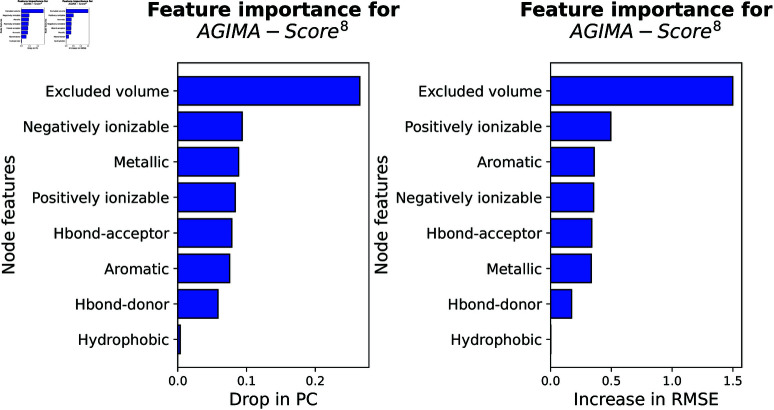
Importance assessment of the node features involved in the AGIMA-Score^8^ model. The result was revealed by masking-based performance drop on the validation set (*PDBbind Core Set*).

**Fig 10:**
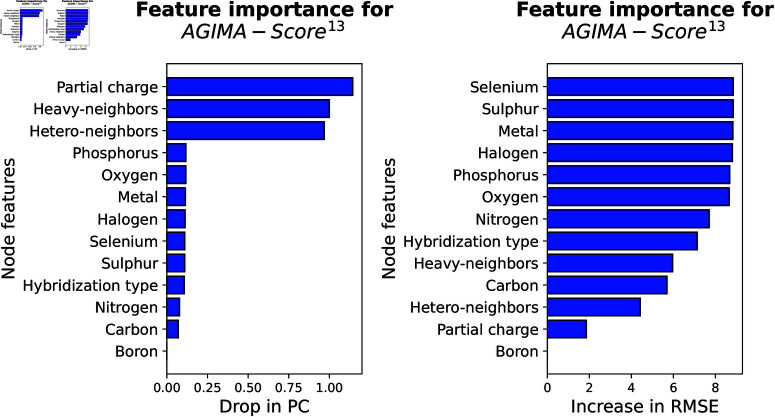
Importance assessment of the node features involved in the AGIMA-Score^13^ model. The result was revealed by masking-based performance drop on the validation set (*PDBbind Core Set*).

The assessment result for *AGIMA-Score*^18^ model is now displayed in Fig 8. As shown here, certain pharmacophoric features (e.g. hydrophobicity, hybridization type and ring membership) weigh heavier importance than the atom types (e.g. Carbon, Nitrogen and Oxygen) in the perspective of either PC drop or RMSE increase. It verifies the important role of certain pharmacophoric properties in determining protein-ligand binding, as frequently applied in pharmacophore-based virtual screening [34]. However, other pharmacophoric properties, like hydrogen-bond donors, are of low interest in this scenario. The *AGIMA-Score*^8^ model depends on a feature set that combines pharmacophoric properties, atomic charges and excluded volume (Fig 9). In this scenario, the excluded volume and atomic charges (positive or negative) stand out from the crowd of pharmacophoric features. The *AGIMA-Score*^13^ model employs a simplified feature set of that from *AGIMA-Score*^18^ (Fig 10). Here the partial charge, heavy-atom neighbors and hetero-atom neighbors dominate the PC drop, while atom types are more important in terms of RMSE increase. In summary of the importance plots, atom features such as certain pharmacophoric properties, atomic charges and connections play a vital role in revealing protein-ligand binding.

**Fig 11:**
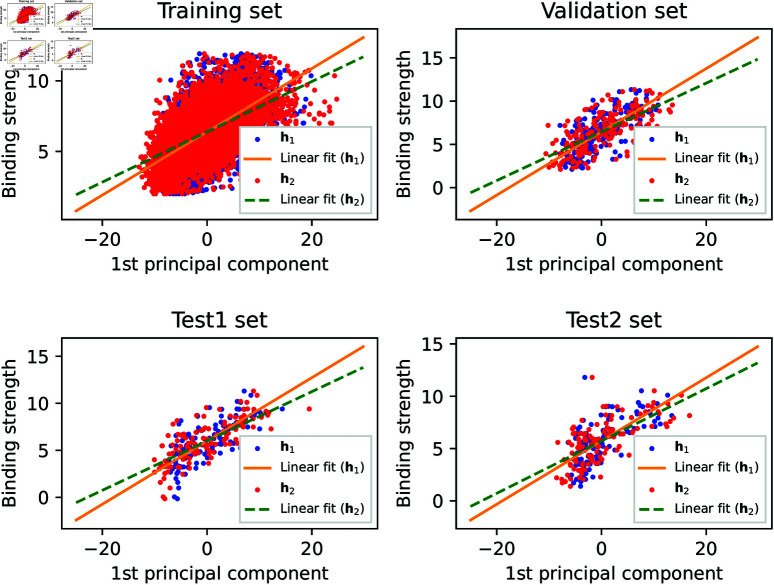
Investigation of key feature embeddings in the AGIMA-Score^8^ model. The feature embeddings ${𝐡}_{i}$ (i=1,2) in the last-but-two layer of the model architecture were decoded by principal component analysis, and the first principal components of ${𝐡}_{i}$ were correlated with the binding strength via linear regression.

***PCA of key feature embeddings.*** The feature embeddings (${𝐡}_{1}$ and ${𝐡}_{2}$) in the last-but-two layer in Fig 3 were monitored in this study, because these hidden features stand for the important molecular interactions learned by an *AGIMA-Score* model. ${𝐡}_{1}$ and ${𝐡}_{2}$ correspond to the inter-molecular interactions in distance ranges 2Å~3Å and 3Å~4Å respectively. PCA was adopted to compress these embeddings, and explore their correlations with the molecular binding strength. In order for better visualization, the first principal component (PC1) for ${𝐡}_{1}$ and that for ${𝐡}_{2}$, of all the complexes in each set were extracted. Examining the correlations between such a PC (of ${𝐡}_{1}$ or ${𝐡}_{2}$) and the binding strength of a complex can provide useful insights into the logics of *AGIMA-Score* models. Taking *AGIMA-Score*^8^ as an example, the *PC1 vs. binding strength* plots for the *Training*, *Validation*, *Test1* and *Test2* sets are now shown in Fig 11. The linear trend for ${𝐡}_{1}$*-PC1 vs. binding strength* and that for ${𝐡}_{2}$*-PC1 vs. binding strengths* were also captured in this figure, where a marked difference in the two trendlines can be observed. This demonstrates two different types of interactions in the process of determining the protein-ligand binding strength.

**Fig 12:**
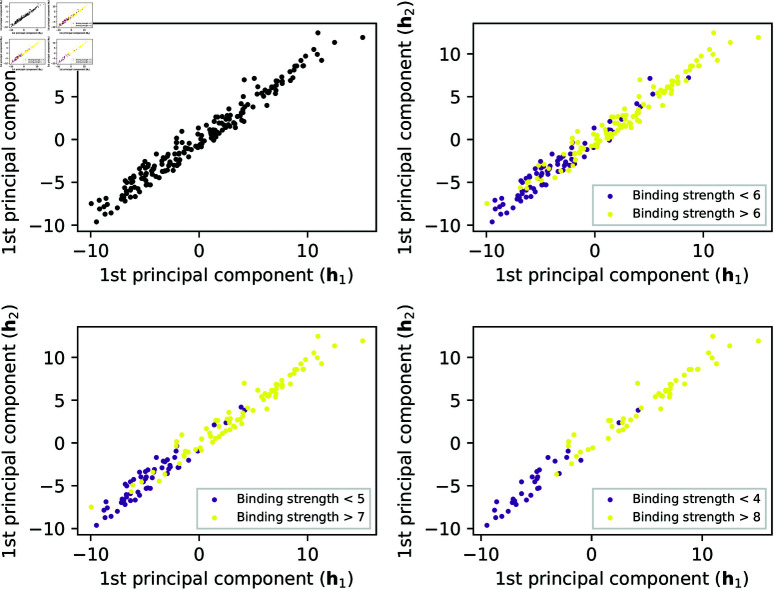
Principal component plots of feature embeddings in the AGIMA-Score^8^ model. ${𝐡}_{1}$-PC1 vs. ${𝐡}_{2}$-PC1 plots for the validation set are shown. Different thresholds of binding strength were used to uncover the correlations between the PCs and the binding strength.

Focusing on the *Validation* set, the scatter plots for the ${𝐡}_{1}$-PC1 and ${𝐡}_{2}$-PC1 of this model are displayed in Fig 12, where multiple thresholds for binding strength are also set to reveal the trends. It shows that higher values of interactions (represented by ${𝐡}_{1}$-PC1 and ${𝐡}_{2}$-PC1) normally lead to higher binding strengths. A similar analysis for *AGIMA-Score*^13^ can be found in S3 and S4 Figs.

## Conclusion

The *AGIMA-Score* framework is introduced in this work. It describes a protein-ligand binding structure as an atomic-level graph, with only the inter-molecular interactions taken into consideration. Having a high computational efficiency, this framework places an absolute focus on the learning of the binding area of a protein-ligand complex. Depending on different sets of node features and a neat graph-learning architecture, a number of *AGIMA-Score* models were constructed. Such models perform well in the scoring of protein-ligand binding strengths and the screening of binders from non-binders for a target protein. At last, they can be explained reasonably from the model level, or in a post-hoc analysis. In the near future, our research will focus on exploring enriched sets of node features and developing more comprehensive approaches for model interpretability.

## Supporting information

S1 FigThe screening performance of each model on the EGFR set, with the top 5~8% ranked ligands considered.In each scenario, the enrichment factors (EFs) regarding various decoy-to-active ratios (*r*_*DTA*_) were calculated for each model, and plotted in a line. The black dashed lines indicate EF = 1(EPS)

S2 FigThe screening performance of *AGIMA-Score* and competing models on the *HIVPR*, *ADA17* and *SRC* sets, with the top 3~4% ranked ligands considered.In each scenario, the enrichment factors (EFs) regarding various decoy-to-active ratios (*r*_*DTA*_) were calculated for each model, and plotted in a line. The black dashed lines indicate EF = 1.(EPS)

S3 FigInvestigation of key feature embeddings in the *AGIMA-Score*^13^ model.The feature embeddings ${𝐡}_{i}$ (i=1,2) in the last-but-two layer of the model architecture were decoded by principal component analysis, and the first principal components of ${𝐡}_{i}$ were correlated with the binding strength via linear regression.(EPS)

S4 FigPrincipal component plots of feature embeddings in the *AGIMA-Score*^13^ model.${𝐡}_{1}$-PC1 vs. ${𝐡}_{2}$-PC1 plots for the validation set are shown. Different thresholds of binding strength were used to uncover the correlations between the PCs and the binding strength.(EPS)

## References

[1] Du-Harpur X, Watt FM, Luscombe NM, Lynch MD. What is AI? Applications of artificial intelligence to dermatology. Br J Dermatol. 2020;183(3):423–30. doi: 10.1111/bjd.18880 31960407 PMC7497072

[2] Johnson KB, Wei W-Q, Weeraratne D, Frisse ME, Misulis K, Rhee K, et al. Precision medicine, AI, and the future of personalized health care. Clin Transl Sci. 2021;14(1):86–93. doi: 10.1111/cts.12884 32961010 PMC7877825

[3] Ivanenkov YA, Polykovskiy D, Bezrukov D, Zagribelnyy B, Aladinskiy V, Kamya P, et al. Chemistry42: an AI-driven platform for molecular design and optimization. J Chem Inf Model. 2023;63(3):695–701. doi: 10.1021/acs.jcim.2c01191 36728505 PMC9930109

[4] Jayatunga MKP, Xie W, Ruder L, Schulze U, Meier C. AI in small-molecule drug discovery: a coming wave?. Nat Rev Drug Discov. 2022;21(3):175–6. doi: 10.1038/d41573-022-00025-1 35132242

[5] Wang DD, Chan M-T. Protein-ligand binding affinity prediction based on profiles of intermolecular contacts. Comput Struct Biotechnol J. 2022;20:1088–96. doi: 10.1016/j.csbj.2022.02.004 35317230 PMC8902473

[6] Wang DD, Xie H, Yan H. Proteo-chemometrics interaction fingerprints of protein-ligand complexes predict binding affinity. Bioinformatics. 2021;37(17):2570–9. doi: 10.1093/bioinformatics/btab132 33650636

[7] Wang DD, Zhu M, Yan H. Computationally predicting binding affinity in protein-ligand complexes: free energy-based simulations and machine learning-based scoring functions. Brief Bioinform. 2021;22(3):bbaa107. doi: 10.1093/bib/bbaa107 32591817

[8] Ballester PJ, Mitchell JBO. A machine learning approach to predicting protein-ligand binding affinity with applications to molecular docking. Bioinformatics. 2010;26(9):1169–75. doi: 10.1093/bioinformatics/btq112 20236947 PMC3524828

[9] Liu Q, Kwoh CK, Li J. Binding affinity prediction for protein-ligand complexes based on β contacts and B factor. J Chem Inf Model. 2013;53(11):3076–85. doi: 10.1021/ci400450h 24191692

[10] Zilian D, Sotriffer CA. SFCscore(RF): a random forest-based scoring function for improved affinity prediction of protein-ligand complexes. J Chem Inf Model. 2013;53(8):1923–33. doi: 10.1021/ci400120b 23705795

[11] Jumper J, Evans R, Pritzel A, Green T, Figurnov M, Ronneberger O, et al. Highly accurate protein structure prediction with AlphaFold. Nature. 2021;596(7873):583–9. doi: 10.1038/s41586-021-03819-2 34265844 PMC8371605

[12] Brown T, Mann B, Ryder N, Subbiah M, Kaplan J, Dhariwal P, et al. Language models are few-shot learners. In: Advances in Neural Information Processing Systems. 2020. p. 1877–901.

[13] Jiménez J, Škalič M, Martínez-Rosell G, De Fabritiis G. KDEEP: protein-ligand absolute binding affinity prediction via 3D-convolutional neural networks. J Chem Inf Model. 2018;58(2):287–96. doi: 10.1021/acs.jcim.7b00650 29309725

[14] Stepniewska-Dziubinska MM, Zielenkiewicz P, Siedlecki P. Development and evaluation of a deep learning model for protein-ligand binding affinity prediction. Bioinformatics. 2018;34(21):3666–74. doi: 10.1093/bioinformatics/bty374 29757353 PMC6198856

[15] Rezaei MA, Li Y, Wu D, Li X, Li C. Deep learning in drug design: protein-ligand binding affinity prediction. IEEE/ACM Trans Comput Biol Bioinform. 2022;19(1):407–17. doi: 10.1109/TCBB.2020.3046945 33360998 PMC8942327

[16] Wang DD, Chan M-T, Yan H. Structure-based protein-ligand interaction fingerprints for binding affinity prediction. Comput Struct Biotechnol J. 2021;19:6291–300. doi: 10.1016/j.csbj.2021.11.018 34900139 PMC8637032

[17] Wang D, Wang R. Scoring protein-ligand complex structures by hybridnet. In: 2023 IEEE International Conference on Systems, Man, and Cybernetics (SMC). 2023. p. 4070–5.

[18] Zheng L, Fan J, Mu Y. OnionNet: a multiple-layer intermolecular-contact-based convolutional neural network for protein-ligand binding affinity prediction. ACS Omega. 2019;4(14):15956–65. doi: 10.1021/acsomega.9b01997 31592466 PMC6776976

[19] Wang DD, Wu W, Wang R. Structure-based, deep-learning models for protein-ligand binding affinity prediction. J Cheminform. 2024;16(1):2. doi: 10.1186/s13321-023-00795-9 38173000 PMC10765576

[20] Son J, Kim D. Development of a graph convolutional neural network model for efficient prediction of protein-ligand binding affinities. PLoS One. 2021;16(4):e0249404. doi: 10.1371/journal.pone.0249404 33831016 PMC8031450

[21] Shen H, Zhang Y, Zheng C, Wang B, Chen P. A cascade graph convolutional network for predicting protein-ligand binding affinity. Int J Mol Sci. 2021;22(8):4023. doi: 10.3390/ijms22084023 33919681 PMC8070477

[22] Zhang X, Gao H, Wang H, Chen Z, Zhang Z, Chen X, et al. Planet: a multi-objective graph neural network model for protein–ligand binding affinity prediction. J Chem Inf Model. 2023. 10.1021/acs.jcim.3c00253 37319418

[23] Wang K, Zhou R, Tang J, Li M. GraphscoreDTA: optimized graph neural network for protein-ligand binding affinity prediction. Bioinformatics. 2023;39(6):btad340. doi: 10.1093/bioinformatics/btad340 37225408 PMC10243863

[24] Feinberg EN, Sur D, Wu Z, Husic BE, Mai H, Li Y, et al. PotentialNet for molecular property prediction. ACS Cent Sci. 2018;4(11):1520–30. doi: 10.1021/acscentsci.8b00507 30555904 PMC6276035

[25] Gilmer J, Schoenholz S, Riley P, Vinyals O, Dahl G. Neural message passing for quantum chemistry. In: International Conference on Machine Learning. 2017. p. 1263–72.

[26] Kipf T, Welling M. Semi-supervised classification with graph convolutional networks. arXiv preprint 2016. https://arxiv.org/abs/1609.02907

[27] Gomes J, Ramsundar B, Feinberg E, Pande V. Atomic convolutional networks for predicting protein-ligand binding affinity. arXiv preprint 2017. https://arxiv.org/abs/1703.10603

[28] Su M, Yang Q, Du Y, Feng G, Liu Z, Li Y, et al. Comparative assessment of scoring functions: the CASF-2016 update. J Chem Inf Model. 2019;59(2):895–913. doi: 10.1021/acs.jcim.8b00545 30481020

[29] Liu Z, Li Y, Han L, Li J, Liu J, Zhao Z, et al. PDB-wide collection of binding data: current status of the PDBbind database. Bioinformatics. 2015;31(3):405–12. doi: 10.1093/bioinformatics/btu626 25301850

[30] Dunbar JB Jr, Smith RD, Damm-Ganamet KL, Ahmed A, Esposito EX, Delproposto J, et al. CSAR data set release 2012: ligands, affinities, complexes, and docking decoys. J Chem Inf Model. 2013;53(8):1842–52. doi: 10.1021/ci4000486 23617227 PMC3753885

[31] Jeffrey G, Jeffrey G. An introduction to hydrogen bonding. New York: Oxford University Press. 1997.

[32] Huey R, Morris G, Forli S, . Using autodock 4 and autodock vina with autodocktools: a tutorial. Scripps Res Inst Molecul Graph Lab. 2012;10550:1000.

[33] Friesner RA, Banks JL, Murphy RB, Halgren TA, Klicic JJ, Mainz DT, et al. Glide: a new approach for rapid, accurate docking and scoring. 1. Method and assessment of docking accuracy. J Med Chem. 2004;47(7):1739–49. doi: 10.1021/jm0306430 15027865

[34] Horvath D. Pharmacophore-based virtual screening. Chemoinformatics And Computational Chemical Biology. 2011. p. 261–98. 10.1007/978-1-60761-839-3_11 20838973

