# Supplementary material for: Scoring protein-ligand binding structures through learning atomic graphs with inter-molecular adjacency
Source: PLoS Comput Biol. 2025 May 9;21(5):e1013074. doi: 10.1371/journal.pcbi.1013074 (PMC12091896; doi:10.1371/journal.pcbi.1013074)
Supplement: Attachment — Submitted filename: ResponseLetter.pdf [file pcbi.1013074.s005.pdf]

Response Letter

We would like to thank the editor and reviewers for their helpful comments. We have revised the manuscript according to their suggestions and provided the point-by-point responses as follows.

Reviewer 1

This paper aims to address the problem of predicting protein-ligand binding strength using a graph-based deep learning model. It represents interacting atoms as graph nodes and interactions as graph edges within two specific distance ranges. The study demonstrates competitive performance in scoring and screening tasks. Model interpretation reveals that features such as pharmacophoric properties play a vital role in complex scoring. Overall, this paper is well-written, and the analysis is comprehensive. However, I still have some concerns that need to be addressed:

1. (*Major*) The use of atomic graphs to represent protein-ligand interactions is well-justified. However, the choice of multi-level distance-dependent adjacency matrices (2-3 Å and 3-4 Å) needs further explanation. Why not use a single matrix (2-4 Å)? If there is a well-known reason for choosing multi-level adjacency matrices over a single one, please ignore it. I observe a strong correlation between h1 and h2 (Figure 9), indicating that h1 and h2 might convey similar features, or multicollinearity. Thus, I think multi-level adjacency might not be necessary. Please explain or discuss this.

Response: Thanks for this suggestion. To investigate whether a single distance range (2-4Å) can cover sufficient adjacency information, we built a single-matrix adjacency tensor $\boldsymbol{A}_{SAM}$ for each complex and constructed three new models by combining $\boldsymbol{A}_{SAM}$ with the three node feature matrices. The three models ($AGIMA-Score_{SAM}^{18}$, $AGIMA-Score_{SAM}^{8}$ and $AGIMA-Score_{SAM}^{13}$) differ from the original models ($AGIMA-Score^{18}$, $AGIMA-Score^{8}$ and $AGIMA-Score^{13}$) only in adjacency matrices, with all the other settings remaining the same. The scoring performances of these three new models have been listed in Table 2 (as below). Although using a single adjacency matrix ($AGIMA-Score_{SAM}$) eases the computations in graph learning, it often results in an underperformance in terms of PC and RMSE, especially when more node features are involved ($AGIMA-Score_{SAM}^{18}$ and $AGIMA-Score_{SAM}^{13}$). It shows that using two adjacency matrices captures more information about the connections among atoms, such as the strong and weak hydrogen bonds with donor-acceptor distances of 2.2-2.5Å and 3.2-4.0Å separately [1]. More details can be found in the *Experiment and Results - Scoring Performance of Models* Section of the revised manuscript with highlights (Pages 8~9).

References:

[1] Jeffrey, George A., and George A. Jeffrey. *An introduction to hydrogen bonding*. Vol. 12. New York: Oxford university press, 1997.

Table 2. Scoring Performance Comparison. The models were trained on *PDBbind Refined Set* (latest version *V2020*) with parameters tuned via the *Core Set* (latest version *V2020*), and tested on two sets from the *CSAR* source. State-of-the-art deep learning models (*ACNN*, *OnionNet*, *KDEEP* and *GraphBAR*) for scoring the protein-ligand complexes were realized, to comprehensively evaluate the proposed *AGIMA-Score* models. For *GraphBAR*, different graph adjacency schemes (2 or 3 adjacency matrices) were adopted for model construction. For *AGIMA-Score*, different node features (separately referring to *Pafnucy*, *KDEEP* and *GraphBAR*) and adjacency schemes (2 adjacency matrices or single adjacency matrix) were considered for model investigation. By default, 2 adjacency matrices (generated by intermolecular atomic contacts within $(2\mathring{A}, 3\mathring{A})$ and those within $(3\mathring{A}, 4\mathring{A})$) were adopted in the graph learning by *AGIMA-Score*. Best performances in terms of PC and RMSE were underlined for the state-of-the-art methods and the proposed *AGIMA-Score* models.

Model	Training		Validation		Test1		Test2	
	PC	RMSE	PC	RMSE	PC	RMSE	PC	RMSE
ACNN	0.5189	1.7564	0.5692	1.7939	0.5596	1.9749	0.6804	1.6298
OnionNet	0.7851	1.2607	0.7843	1.4807	0.6365	1.8011	0.6123	1.7329
KDEEP	0.9137	1.0243	0.8887	1.1773	0.604	1.9077	0.7169	1.6534
$GraphBAR_{2AM}$	0.6102	1.5664	0.6367	1.7893	0.6425	1.8055	0.7149	1.6003
$GraphBAR_{3AM}$	0.6206	1.6785	0.6500	1.9082	0.6526	1.9140	0.7133	1.7137
$AGIMA\text{-}Score^{18*}$	0.6543	1.5000	0.7089	1.6858	0.6773	1.7423	0.7295	1.5778
$AGIMA\text{-}Score^{18*}_{SAM}$	0.6338	1.5131	0.6579	1.7344	0.6302	1.8062	0.6911	1.6090
$AGIMA\text{-}Score^{8+}$	0.5526	1.6175	0.6582	1.7251	0.6464	1.8009	0.7090	1.6120
$AGIMA\text{-}Score^{8+}_{SAM}$	0.5440	1.6283	0.6422	1.7485	0.6493	1.8020	0.7094	1.6072
$AGIMA\text{-}Score^{13@}$	0.6251	1.5389	0.6913	1.6316	0.6794	1.7089	0.7150	1.5838
$AGIMA\text{-}Score^{13@}_{SAM}$	0.6106	1.5467	0.6420	1.7555	0.6582	1.7623	0.7117	1.5867
$AGIMA\text{-}Score^{21\#}$	0.6137	1.5538	0.6754	1.6659	0.6651	1.7274	0.7272	1.5544

2. (*Major*) Model Comparison: What are the key differences between the GraphBAR and AGIMA-Score 13 models? Both models appear to use 13 features and two adjacency matrices, yet AGIMA-Score 13 performs much better (e.g., in test set 1, 0.6463 vs. 0.6794). Please discuss the key model improvements and possible reasons.

Response: The major difference between *GraphBAR* and $AGIMA - Score$ lies in the atomic adjacencies in the graph learning. *GraphBAR* considers both intra- and inter-molecular contacts (Figure 2B as below) while $AGIMA - Score$ focuses on only the inter-molecular contacts (Figure 2D as below) in the construction of molecular graphs. Emphasizing the inter-molecular adjacencies not only eases the computations but also places a focus on the binding area, which will benefit tasks like binding-strength scoring.

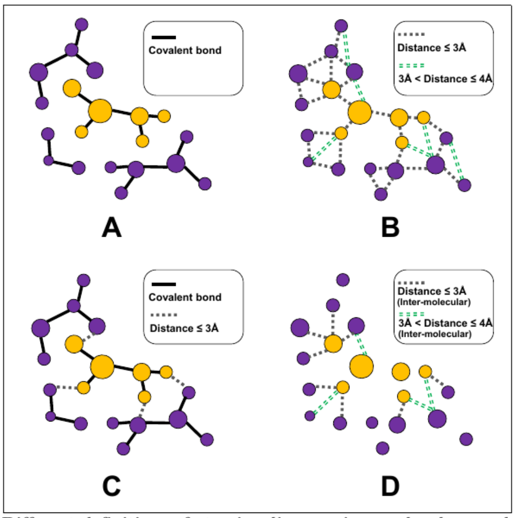

Fig 2. Different definitions of atomic adjacency in a molecular graph. A. Covalent adjacency. **B**. Distance-dependent contacts. **C**. A combination of covalent adjacency and distance-dependent contacts. **D**. Inter-molecular contacts through distance thresholding.

To make a fair comparison, we constructed two $GraphBAR$ models in the revised manuscript ($GraphBAR_{2AM}$ and $GraphBAR_{3AM}$), using the same learning architecture as $AGIMA-Score$. $GraphBAR_{2AM}$ takes into account the intra-/inter-molecular contacts within 0-2Å (mostly intra-molecular) and those within 2-4Å (intra- and inter-molecular) when building the adjacency tensor, which corresponds to the $AGIMA-Score_{SAM}$ models (considering only inter-molecular contacts in 2-4Å). $GraphBAR_{3AM}$ adopts three distance ranges (0-2Å, 2-3Å and 3-4Å) to collect the intra-/inter-moleclar adjacencies, corresponding to the $AGIMA-Score$ models (considering only inter-molecular contacts in 2-3Å and 3-4Å). As shown in Table 2 (Page 9 of the revised manuscript), $GraphBAR$ models often result in an underperformance compared to $AGIMA-Score$ models. It may demonstrate the efficacy of decoding the inter-molecular contacts by simple graph-learning architectures in a binding-strength scoring task. More details can be found in the *Experiment and Results - Scoring Performance of Models* Section of the revised manuscript with highlights (Pages 8~9).

3. (*Minor*) More features (e.g., 13 features) generally lead to better performance, and the paper also suggests it in both scoring and screening tasks. Naturally, I am wondering why the author didn' t combine those non-redundant features to train a single model. I have also seen language model embeddings used as additional features. The author might want to try it (no need for this paper). However, you might need to discuss feature selection for future model development based on your model interpretation.

Response: For the three node-feature matrices, $\mathbf{F}^{18}$ includes generic physico-chemical properties and pharmacophoric properties of atoms, $\mathbf{F}^{8}$ focuses on pharmacophoric properties while $\mathbf{F}^{13}$ emphasizes generic physico-chemical properties. In addition, $\mathbf{F}^{13}$ is a subset of $\mathbf{F}^{18}$, and $\mathbf{F}^{8}$ overlaps with $\mathbf{F}^{18}$ largely. We have provided the information of these node feature sets in Table 1 of the *Experiment and Results* Section (Pages 6~8).

As an additional experiment, we have collected the non-redundant features $\mathbf{F}^{21}$ from $\mathbf{F}^{18}\cup\mathbf{F}^{8}\cup\mathbf{F}^{13}$, and constructed a new model $AGIMA-Score^{21}$ for comparison purposes. As shown in Table 2 (Page 9 of revised manuscript), the performance of $AGIMA-Score^{21}$ is no better than $AGIMA-Score^{18}$, implying that $\mathbf{F}^{21}$ and $\mathbf{F}^{18}$ cover similar node information. Please refer to the revised manuscript with highlights for more information (*Experiment and Results - Scoring Performance of Models* Section, Pages 8~9).

Reviewer 2

The authors present AGIMA (Atomic Graphs with Inter-Molecular Adjacency), a graph-based method developed to predict binding affinities of protein-ligand complexes. The method also has screening capabilities, i.e., it can assign higher ranks to the true binders of the target protein. Predicting the binding affinity of protein-ligand complexes is a very relevant question in structural biology. The field has experienced significant developments in predicting three-dimensional protein and protein complex structures in the last few years. However, binding affinity prediction is still an open question that needs to be solved. New computational methods are needed, and if successful, they will have a great impact on drug discovery and biomedical research. AGIMA represents the protein-ligand complex as a graph where nodes are the atoms of the binding interface characterized by physico-chemical properties, and edges are defined by an adjacency tensor that handles two different cutoff distances to define inter-molecular contacts. A Chebnet Graph Neural Network (GNN) is used to learn from the constructed graphs. Unlike other methods, AGIMA focuses only on inter-molecular interactions involving atoms of the binding interface. Additionally, it applies two different distance ranges (2-3 A and 3-4 A) to define these inter-molecular interactions. The authors used the Refined (4856 cases) and Core (285 cases) Sets of the PDBbind database for training and validating the model, respectively. Two independent sets from the CSAR database, with 175 and 167 protein-ligand complexes, were used for testing. Additionally, a specific set of epidermal growth factor receptor (EGFR) protein-ligand structures from the DUD-E database was used to evaluate the models' screening capabilities. AGIMA is compared with four other deep learning-based methods and shows a competitive performance. The article is easy to read and well-structured. However, I suggest a review to correct some typos and the legibility of several sentences.

Response: Thank you for the comment. We have reviewed the manuscript carefully to correct the typos and refine the presentation.

Additionally, I would propose the following suggestions for consideration:

1. (*Major*) One of the most critical elements when developing machine learning methods is having optimal datasets for training, validation and testing. Deep learning algorithms, in particular, are very demanding and usually require a large and diverse dataset during training. One main concern about this work is related to this aspect. The authors have used a dataset of 4856 instances to train the model. It is not clear that such a small dataset can be representative of the diversity of the protein-ligand complex conformational space and effectively used to train the model. An analysis of the dataset's characteristics would be helpful to address these concerns. What are the protein families represented in the datasets? What is the protein sequence variability within and between datasets? What is the variability of the ligands? The authors mention that there is no overlap between the training and test sets, but how this was enforced needs to be clarified. For example, a good

approach to test the model's generalization capabilities would be to ensure that the proteins in the training and test sets do not share a sequence identity above a given threshold (e.g. 20-30%).

Response: Many thanks for this valuable comment. The *PDBbind* database (latest version: V2020) [1] was curated by filtering and integrating data from several sources, such as Protein Data Bank (PDB), literature and ChEMBL. It aims to connect protein-ligand *complex structures* (experimentally determined) with the *binding affinity data* (experimentally measured). The developers of *PDBbind* have further generated the *Refined Set*, which contains high-quality protein-ligand complexes with refined structures and accurate binding affinity data [2]. The filtering criteria for this set include

(1) Complex structures should have a resolution of <= 2 Å to ensure well-defined structures.
(2) Ligands should have a molecular weight no larger than 1000Da to guarantee small drug-like ligands.
(3) Complexes should have high-quality, experimentally measured binding affinity data (e.g. in $K_{d/i}$ format).
(4) Complexes should share a sequence identity of no more than 30% to avoid redundancy.
(5) Ligands should have a similarity of no more than 70% to ensure ligand diversity.

The *Refined Set* (1,486 unique proteins and 5,316 complexes) covers a diverse set of protein families (e.g. enzymes, receptors, transporters and others), and it has been widely acknowledged as a benchmark dataset for evaluating computational methods that predict protein-ligand binding strengths. The validation (285 complexes) and test sets (176 and 167 complexes) are mostly from this *Refined Set*, and they have been broadly adopted as validation/test sets in previous studies [3-6]. To guarantee a credible evaluation, we have ensured that the overlapped complexes have been removed from the *Refined Set* before model training (remaining 4856 complexes). We have briefly described the datasets in the revised manuscript, and more details can be found in *Experiment and Results - Scoring Performance of Models* Section of the revised manuscript with highlights (Pages 8~9).

References:

[1] Liu, Zhihai, Yan Li, Li Han, Jie Li, Jie Liu, Zhixiong Zhao, Wei Nie, Yuchen Liu, and Renxiao Wang. "PDB-wide collection of binding data: current status of the PDBbind database." *Bioinformatics* 31, no. 3 (2015): 405-412.

[2] Su, Minyi, Qifan Yang, Yu Du, Guoqin Feng, Zhihai Liu, Yan Li, and Renxiao Wang. "Comparative assessment of scoring functions: the CASF-2016 update." *Journal of chemical information and modeling* 59, no. 2 (2018): 895-913.

[3] Gomes, Joseph, Bharath Ramsundar, Evan N. Feinberg, and Vijay S. Pande. "Atomic convolutional networks for predicting protein-ligand binding affinity." *arXiv preprint arXiv:1703.10603* (2017).

[4] Jiménez, José, Miha Skalic, Gerard Martinez-Rosell, and Gianni De Fabritiis. "K deep: protein–ligand absolute binding affinity prediction via 3d-convolutional neural networks." *Journal of chemical information and modeling* 58, no. 2 (2018): 287-296.

[5] Zheng, Liangzhen, Jingrong Fan, and Yuguang Mu. "Onionnet: a multiple-layer intermolecular-contact-based convolutional neural network for protein–ligand binding affinity prediction." *ACS omega* 4, no. 14 (2019): 15956-15965.

[6] Son, Jeongtae, and Dongsup Kim. "Development of a graph convolutional neural network model for efficient prediction of protein-ligand binding affinities." *PloS one* 16, no. 4 (2021): e0249404.

2. (*Major*) A similar concern applies to the dataset used to evaluate the model's screening capabilities. The dataset only contains complexes for a single protein, the EGFR protein. How similar is EGFR to the proteins of the training set? In any case, I would suggest that a dataset containing more proteins should be used for this task to guarantee a good evaluation of the model's performance.

Response: Thanks for this comment, and more background information on such tasks has been given in the revised manuscript to provide a better understanding to the readers. *Virtual screening* aims to discover the potential binders for a target protein, in order to mitigate the burden of downstream biochemical experiments (Figure 4 below). Such a target protein often plays a key role in regulating the progression of some diseases, such as the epidermal growth factor receptor (EGFR) protein that influences the growth of non-small-cell lung cancer (NSCLC). We usually score the protein-ligand binding structures and rank the scores to determine whether the true binders for the target protein can be highly ranked. This is regarded as a main indicator of the screening capability of the employed scoring model.

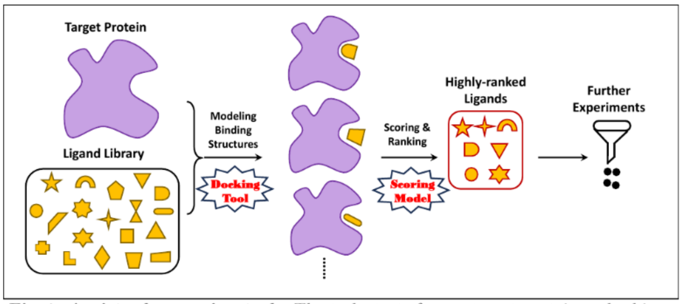

Fig 4. A virtual screening task. The task starts from a target protein and a big library of ligands, followed by the modeling of each protein-ligand binding structure (docking tool) and the scoring of the binding structures (scoring model). The highly-ranked ligands (according to the predicted scores) will be regarded as potential binders for further biochemical experiments.

In order to provide a more comprehensive evaluation, two more screening tasks, involving the HIVPR and ADA17 proteins, are presented in the revised manuscript. The best screening performances of the $AGIMA-Score$ models on these two sets coupled with the EGFR set are displayed in Figure 6. When considering all the decoys in the screening tasks, these models have achieved good EFs of 13, 33 and 16 for the top 1% ranked ligands of the three sets. Detailed information has been provided in the revised manuscript with highlights (*Experiment and Results - Screening Performance of Models* Section, Pages 10~12).

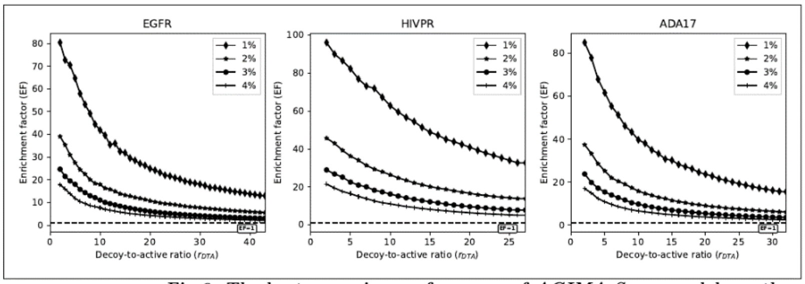

Fig 6. The best screening performance of *AGIMA-Score* models on the *EGFR*, *HIVPR* and *ADA17* sets, with the top $1 \sim 4\%$ ranked ligands considered. In each scenario, the enrichment factors (EFs) regarding various decoy-to-active ratios (r_{DTA}) were calculated for each model, and plotted in a line. The black dashed lines indicate EF=1.

3. (*Major*) Authors indicate the datasets used in their work. They also include the references to the original sources. As these sources might be subject to new versions, etc., I recommend storing the final datasets used in this work in a public repository lke Zenodo to guarantee the long-term availability of the data and reproducibility of the results. Ideally, the datasets and file structure defined at point "3. Data downloading and preprocessing" of the project Github repository (https://github.com/debbydanwang/DL-PLBAP) should be available at Zenodo or a public repository.

Response: Thanks for this very helpful comment. We have uploaded the datasets (atom properties files for the Refined Set, Core Set and two test sets), which can be used directly for representation learning and model construction, to *Zenodo*. The DOI (https://doi.org/10.5281/zenodo.13352486) has also been provided in the Github repository of this project (screenshot as below).

```
        - For easier access, these atom property files have been
deposited in Zenodo (DOI of https://doi.org/10.5281/zenodo.13352486).
They can be used directly for representation learning and model
construction.
```

4. (*Major*) The method requires the structure of the protein-ligand complex as input. This could be a limitation, as the number of available structures is very small compared with the number of available sequences. Have the authors considered assessing the method's performance using modelled protein-ligand complexes instead of experimental structures? It would be interesting to compare the results of the method using experimental or modelled structures as input. If performance with structural models was good, the method's applicability would increase.

Response: In the screening task regarding a specific target protein and its potential ligands, we do not have the protein-ligand complex structures at the beginning (below Figure 4). (1) The first subtask is therefore modeling the complex structure of each protein-ligand pair. Current state-of-the-art docking tools (e.g. AutoDOCK and Glide) can generate near-experimental complex structures, and we also used such tools for structure generation. (2) Once the modeled complex structures are derived, they can be fed into our scoring models for binding-strength prediction. These predicted scores will then be ranked, with the top ones treated as most potential binders.

As aforementioned, the trained scoring model can handle modeled/generated complex structures. In our work, we adopted the datasets from DUD-E database to evaluate the screening power of our scoring model. In a DUD-E set, the label (binder/non-binder) for each ligand regarding a target protein is given. The evaluation process includes (1) modeling each protein-ligand complex structure, (2) scoring each structure by $AGIMA - Score$, (3) ranking all the ligands based on the predicted scores, and (4) focusing on the highly ranked (e.g. top 1%) ligands (with known labels) to calculate the enrichment factors. Good EFs have been derived in the screening tasks, demonstrating that $AGIMA - Score$ can handle modeled complex structures decently.

More details can be found in the revised manuscript with highlights (*Experiment and Results* - *Screening Performance of Models* Section, Pages 10~12).

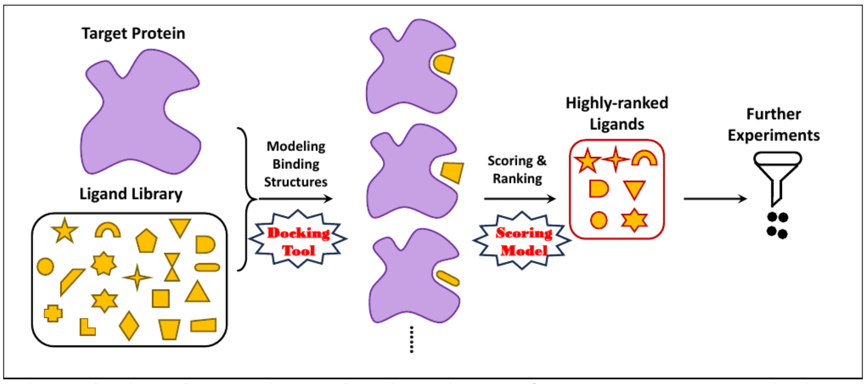

Fig 4. A virtual screening task. The task starts from a target protein and a big library of ligands, followed by the modeling of each protein-ligand binding structure (docking tool) and the scoring of the binding structures (scoring model). The highly-ranked ligands (according to the predicted scores) will be regarded as potential binders for further biochemical experiments.

5. (*Major*) It would be interesting to include in the benchmark methods that do not require the structure of the complex but only the sequences as input.

Response: Thanks for this comment. Our works are mainly focused on *structured-based* scoring, which is structure-oriented and towards more interpretable molecular representations/models. *Sequence-based* models emphasize the prediction efficiency and accuracy more. Comparing these two groups of methods, structure-based works may be limited to the availability of structural data while sequence-based methods may lack interpretability and mechanism-driven knowledge.

As we are not so familiar with sequence-based scoring works, we implemented a simple sequence-based work for your information. For each protein-ligand complex, the protein was represented by the amino-acid sequence and the ligand by the SMILES string. Before learned by a model, these data should be digitized. We transformed the protein sequence by a mapping dictionary and the SMILES string to ECFP (radius of 2 and length of 1024). To standardize the final sequence length, the protein sequence takes 1300 positions and the ligand ECFP occupies 300 positions. The concatenated string (length of 1600) was regarded as the input sequence for each complex, and we adopted one widely-applied, sequence-oriented deep learning technique (RNN) for mapping these sequences to their binding strengths. A simple architecture with one LSTM layer (size of 256), one dense layer (size of 128) and one output layer was adopted in this work. ***A pretty worse result was obtained from this work (test1: PC-0.189/RMSE-5.55, test2: PC-0.157/RMSE-5.24), and maybe more sequence data are required to train a decent sequence-based model.*** Due to the unfamiliarity with sequence-based models and the comparison unfairness caused by it, we did not provide this work in the manuscript. More works will be done for this comparison purpose in the future.

6. (*Major*) To improve clarity and understanding, I would suggest to expand the information provided in the table and figure captions of the paper so they are as self-explanatory as possible.

Response: We have enriched the captions of tables 1~2 and figures 3~6, in order to provide an easier understanding to the readers. These changes can be found in the revised manuscript with highlights.

7. (*Minor*) The Results section includes key information about the datasets used in the work, the sets of features used to characterize the network nodes, and the method applied to evaluate the model's screening performance. I suggest providing this information in the Methods section.

Response: We have provided the information of node feature sets in Table 1 of the *Experiment and Results* Section (Pages 6~8). $\mathbf{F}^{18}$ includes generic physico-chemical properties and pharmacophoric properties of atoms. $\mathbf{F}^{8}$ focuses on pharmacophoric properties while $\mathbf{F}^{13}$ emphasizes generic physico-chemical properties. Please refer to this section in the revised manuscript with highlights for more details.

8. (*Minor*) Could the authors indicate how much time does a prediction require on a standard machine (for example, seconds on a desktop PC or high-performance computing cluster)?

Response: All the experiments were run on a workstation with a single GPU (GeForce RTX 2080 with an 8GB RAM). Training an $AGIMA - Score$ model on the Refined Set, with epochs and batch size mainly tuned, requires ~3 hours. Once we have this trained model, the predictions are quite fast (e.g. less than 1 second for the whole validation/test set). As the structural and binding data grow rapidly, training such a model on a larger set in the future will require more powerful GPUs or computer servers.
