# Supplementary material for: Scoring protein-ligand binding structures through learning atomic graphs with inter-molecular adjacency
Source: PLoS Comput Biol. 2025 May 9;21(5):e1013074. doi: 10.1371/journal.pcbi.1013074 (PMC12091896; doi:10.1371/journal.pcbi.1013074)
Supplement: Attachment — Submitted filename: ResponseLetter_auresp_2.pdf [file pcbi.1013074.s006.pdf]

Response Letter

We would like to thank the editor and reviewers for their helpful comments. We have revised the manuscript according to their suggestions and provided the point-by-point responses as follows.

Reviewer 1

The authors have submitted a comprehensive revision, and I now consider this manuscript ready for publication. My primary concern in the initial submission was the justification for using multi-level distance-dependent adjacency matrices and incorporating all 21 non-redundant features in the final model. The revised approach demonstrates a significant improvement through the use of these adjacency matrices, which is clearly validated against other models. I am now fully satisfied with the authors' justification on this point.
Additionally, the responses to my other revision requests, as well as (in my view) those raised by the other reviewers, are clear, detailed, and comprehensive.:

Response: Thanks for this comment and the previous comments that make our manuscript more credible.

Reviewer 2

First of all, I would like to thank the authors for their responses to my comments. While most of these responses have completely addressed my concerns, I still have a few comments that I believe need further clarification.

1. My initial question highlighted the importance of having a large and diverse training dataset. I suggested conducting an analysis of the dataset characteristics to evaluate these aspects. I also asked about the measures taken to ensure there was no overlap between the training and test sets.

   In their response, the authors state that they have ensured no complexes are shared between the training and test sets. While it is true that the training and test sets do not share complexes with the same PDB ID, I believe that the training and test sets still contain models with different PDB IDs that represent the same complex. For example, the PDBs 2e2r and 2p7g, which are part of test1, represent the same complex (ESTROGEN-RELATED RECEPTOR GAMMA in complex with ligand bisphenol A) as PDB 6i63 in the training set. Similarly, PDB 1ax0 in test2 represents the same complex (ERYTHRINA CORALLODENDRON LECTIN in complex with ligand N-ACETYLGALACTOSAMINE) as PDB 3n35 in the training set. This means that the test sets used to evaluate the model contain complexes that the model has already seen during training, even if the experimental structures have different PDB IDs. To ensure the performance of the model has been evaluated correctly, all

> such instances should be identified and corrected. As the authors suggest in their response, a proper split would be one in which the complexes in the training and test sets do not share more than 30% sequence identity and ligand similarity does not exceed 70%.
>
> The same precautions should be applied to the cases used to assess the potential of the models for virtual screening.

Response: Many thanks for this valuable comment. In our point of view, structure-based predictions count more on the ***structural difference*** rather than ***sequence difference***. Although complexes A and B contain similar or even identical protein sequences and ligands, the actual structures of the complexes may differ (molecules are dynamic objects in a solvent environment). For the two examples raised by the reviewer (2e2r/2p7g vs. 6i63 and 1ax0 vs. 3n35), we have investigated the structural differences for each pair. For easier interpretation, the structural deviations of protein binding pockets for the comparing pairs are displayed (structures aligned and displayed by *UCSF Chimera* software; note that ligand structures may also differ but are not shown here). As shown here, each pair of complexes are structurally different, and such differences result in variations in the binding strengths. The **structural differences** and **binding-strength deviations** make the complexes **different samples** in the prediction task, and we believe this is the reason why the developers of this database (*PDBbind*) regard such complexes as separate samples.

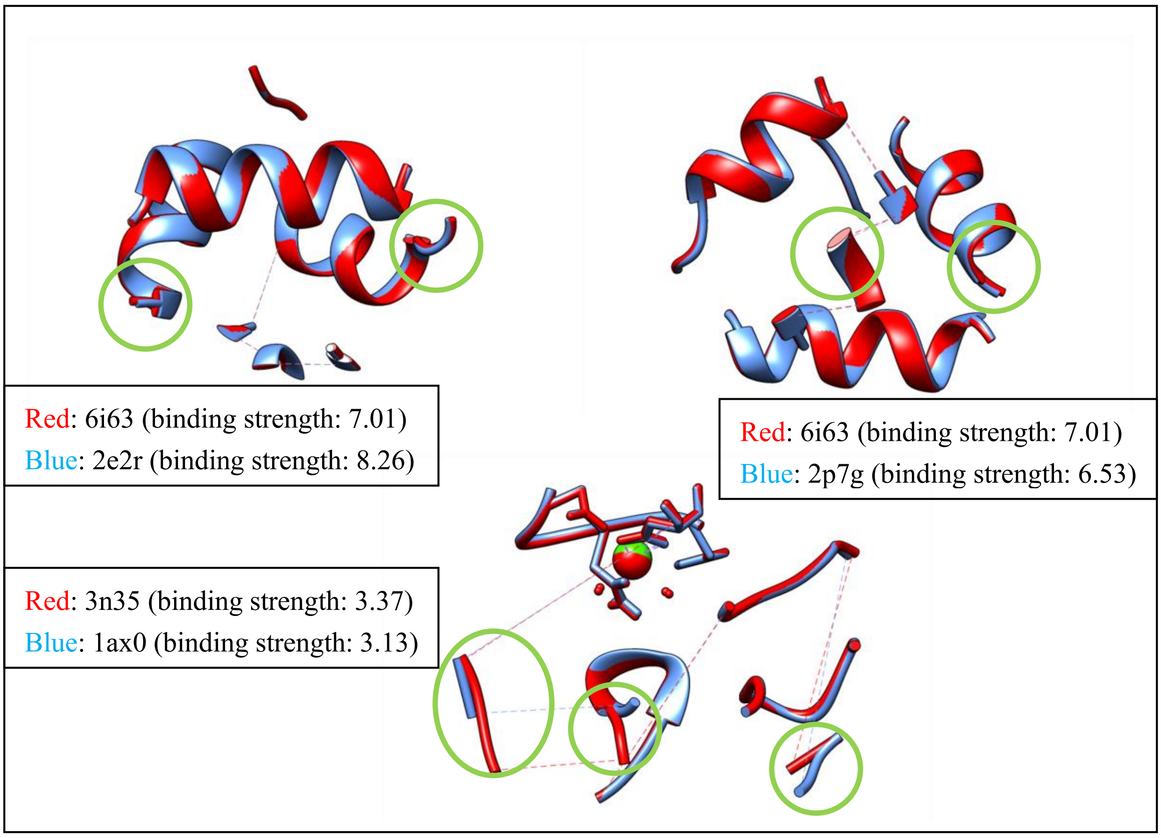

Fig1. Structural comparisons among complexes having similar sequences/ligands.

In a screening task regarding a specific target protein (e.g. EGFR), different ligands bind to the same target in variant poses (position plus orientation), and it makes the binding complexes totally different samples **in term of structures** (unseen from training set).

2. Regarding the diversity of the Refined set from which the training set was generated, the authors state that, during the generation of the Refined set, criteria were applied to ensure that the complexes do not share more than 30% sequence identity, to avoid redundancy, and that ligands do not exceed 70% similarity. However, the Refined set contains multiple instances of the same complex represented by different PDBs. This means that the number of unique complexes in the Refined dataset is lower than the 5,316 distinct PDB IDs it contains. To identify such issues, I suggested that the authors conduct an analysis of the diversity of the proteins and ligands in the datasets used. I believe this analysis remains relevant and would help assess the scope and limitations of the dataset used to train the model.

Response: Thanks for this very insightful comment. According to the suggestion of the reviewer, we have scanned *each pair of complexes* in the *Refined set* (5,316 complexes) of the database. The filtering criterion is that a valid pair of complexes should have a protein-sequence similarity of <30% **OR** a ligand similarity of <70% **OR** different binding strengths. Pairwise protein similarity calculation was implemented using R package '*protr*' (local alignment based on *BLOSUM62* substitution matrix), ligand similarity calculation was done using R package '*ChemmineR*' (Tanimoto coefficient of descriptor vectors), and binding strengths were retrieved from *PDBbind* database. All but 19 pairs of complexes passed the filtering (Fig 2), and we have manually investigated the structural differences of these pairs. Examples showing such structural differences are displayed in Fig 3. Accordingly, we believe that all the complexes in this set can be regarded as **separate, valid samples** from a **structural point of view**.

Complex1	Complex2
5fot	5fox
6d1d	6d1g
2zz1	2zz2
5mkr	5mks
1jvu	1o0m
6ggb	6ggf
1q72	1qyg
5lli	5nvx
2hmu	2hmv
3f15	3nx7
1g48	1i9p
1t7j	2f8g
3i5z	3i60
2idw	3ekp
2qd7	3pwm
6n78	6n79
3djk	3ekt
3i7e	5ult
2i4u	4ll3

Fig 2. Complex pairs do not pass the filtering.

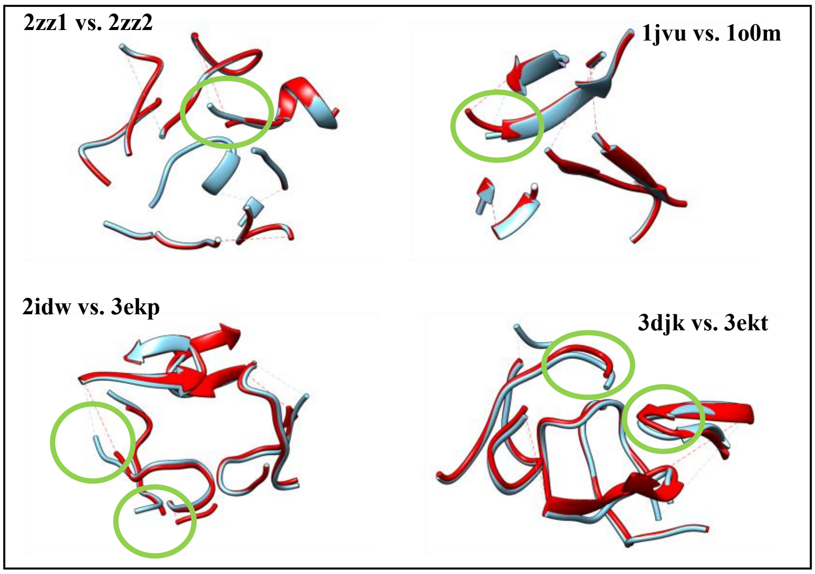

Fig 3. Structural comparisons for complex pairs in Fig 2.

3. If the issues with the training and test sets described above are resolved and the model's performance remains strong, it would be very useful for the scientific community to have access to a Docker or Singularity container, or a server, with a trained AGIMA model available for use.

Response: Thanks for this comment. From a structural perspective, we believe the data we used for testing are unseen from the training data, which guarantees that the reported results are valid and credible. As another verification, a number of existing structure-based scoring functions in Docking tools (e.g. *DrugScore* [1], *ChemScore* [2], *GoldScore* [3] and *GlideScore* [4]) were indeed developed from this *PDBbind* database, and they have been widely acknowledged in this area. A short discussion about the structural and sequential differences of involved complexes has been added to the revised manuscript (Section *Discussion on Structural and Sequential Similarity*). As last, we hope our explanations can address the reviewer's concerns about the data.

References:

[1] Dittrich, Jonas, Denis Schmidt, Christopher Pfleger, and Holger Gohlke. "Converging a knowledge-based scoring function: DrugScore2018." *Journal of chemical information and modeling* 59, no. 1 (2018): 509-521.

[2] Parker, Kenneth C. "Scoring methods in MALDI peptide mass fingerprinting: ChemScore, and the ChemApplex program." *Journal of the American Society for Mass Spectrometry* 13, no. 1 (2002): 22-39.

[3] Verdonk, Marcel L., Jason C. Cole, Michael J. Hartshorn, Christopher W. Murray, and Richard D. Taylor. "Improved protein–ligand docking using GOLD." *Proteins: Structure, Function, and Bioinformatics* 52, no. 4 (2003): 609-623.

[4] Wang, Wei, Xi Zhou, Wanlin He, Yi Fan, Yuzong Chen, and Xin Chen. "The interprotein scoring noises in glide docking scores." *Proteins: Structure, Function, and Bioinformatics* 80, no. 1 (2012): 169-183.
