# Supplementary material for: Scoring protein-ligand binding structures through learning atomic graphs with inter-molecular adjacency
Source: PLoS Comput Biol. 2025 May 9;21(5):e1013074. doi: 10.1371/journal.pcbi.1013074 (PMC12091896; doi:10.1371/journal.pcbi.1013074)
Supplement: Attachment — Submitted filename: ResponseLetter_auresp_3.pdf [file pcbi.1013074.s008.pdf]

Response Letter

We would like to thank the editor and reviewer again for their helpful comments. We have revised the manuscript accordingly and provided the responses as follows.

Reviewer 2

1. Relying on visual or graphical assessments to determine structural similarity can be imprecise and subjective. I conducted an analysis of the structures using Cα-RMSD (C-alpha Root Mean Square Deviation) and obtained the following results:

The Cα-RMSD for the proteins in complexes 6i63 and 2p7g is 0.33 Å, and for 6i63 and 2e2r, it is 0.56 Å. Additionally, the RMSD of the ligands between 6i63 and 2p7g after superposing the proteins is 0.21 Å. For complexes 3n35 and 1ax0, the Cα-RMSD is 0.28 Å, while the RMSD for their ligands is 0.54 Å.

These values are sufficiently low to consider the structures practically identical.

Response: Thanks for this comment. We agree on that those structures are practically identical after careful inspection and literature review. To avoid such potential data leakage, we have done certain similarity analysis and cleaned the data accordingly. Please check the responses to Comments 3 and 4 for more information.

2. As you point out, proteins are flexible molecules that exist in a variety of conformations, often referred to as the "conformational ensemble." Experimental binding strength measurements reflect an average over this ensemble and are not associated with a single static structure. Therefore, I respectfully disagree with the statement that "each pair of complexes are structurally different, and such differences result in variations in the binding strengths." In my opinion, the complex pairs in question are structurally identical and most likely represent identical conformational ensembles. The differences in binding strengths are probably due to experimental errors, varying experimental conditions, or similar factors.

Response: Thanks for this comment. We agree on that those structures are practically identical after careful inspection and literature review. To avoid such potential data leakage, we have done certain similarity analysis and cleaned the data accordingly. Please check the responses to Comments 3 and 4 for more information.

3. I might be repeating myself, but I believe a key issue that has been overlooked in the different versions of the paper is a thorough analysis of the degree of similarity between the training and test sets. Published studies have demonstrated that the similarity between sequences and structures in these sets can significantly impact the performance of machine learning and AI methods for predicting binding strength.

I consider important to include an in-depth analysis of the similarity of both proteins and ligands in the training and test sets. Additionally, studying how the model's

accuracy varies depending on this similarity would provide valuable insights into the generalization power of the model.

Response: Thanks for this comment. To avoid potential train-test contamination, each pair of complexes, one from the validation set and the other from the training set, needs to pass a similarity test. This test guarantees that the similarity of two protein sequences is below 0.3 OR the similarity of two ligands is below 0.7, in each pair of complexes. The complexes against this rule were removed from the validation set. This similarity test was also performed on the two test sets to remove the problematic complexes. After this cleaning, the similarity statistics for pairwise complexes are presented in the figure below (*Figure 4 of the revised manuscript*). The updated PDB lists for Training, Validation, Test1 and Test2 sets are uploaded to our *Zenodo* repository (https://doi.org/10.5281/zenodo.14504532).

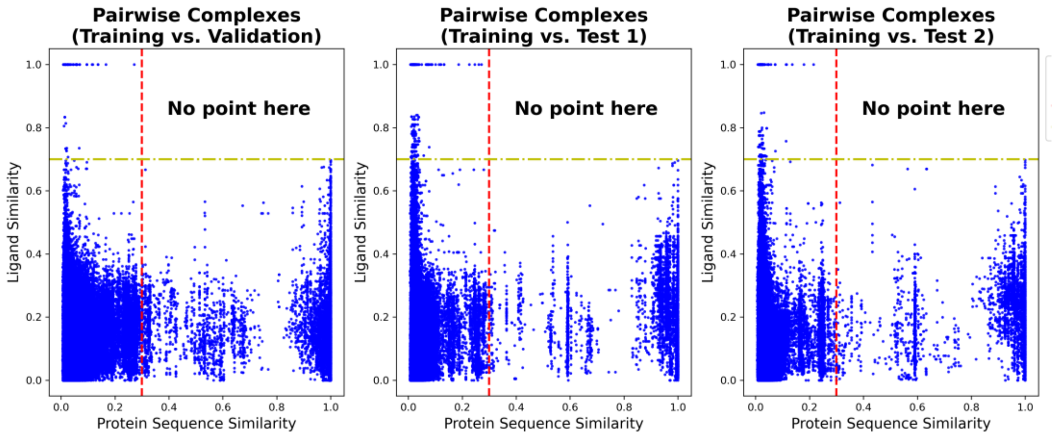

Fig1. Similarity test for each pair of complexes (Training vs. Validation, Training vs. Test1, and Training vs. Test2).

Based on the updated data, we have re-implemented all the experiments, including scoring, screening and post-hoc model analysis. (1) The scoring performances of AGIMA-Scores and the competitors are listed in *Table 2 of the revised manuscript*. Although the performances differ slightly from the earlier version, the superiority of AGIMA-Scores does not change. (2) To further support our studies, we have applied the models to more tasks than the previous version. These tasks concern protein targets (EGFR, HIVPR, ADA17 and SRC) and their ligands (actives and decoys). The screening performances are displayed in *Figures 6~7 in the revised manuscript* and *Figures S1~S2 in the supporting information*. They demonstrate the outperformance of the group of AGIMA-Scores over others. (3) The post-hoc model analysis was updated as well, and the results are now presented in *Figures 8~12 in the revised manuscript* and *Figures S3~S4 in the supporting information*. More details can be found in the revised manuscript with highlighted changes.

4. I would also like to mention a point from my previous review regarding the number of unique complexes in the Refined dataset. I initially noted that the number of unique complexes is lower than the 5,316 distinct PDB IDs it contains. This is relevant because, if the model's generalization capacity is not high, it is important

to have a wide and diverse training set. In response, you ran a protocol to identify valid pairs of complexes, defined as those with protein sequence similarity of less than 30% or ligand similarity of less than 70%. You also included the criterion of "different binding strengths." However, I would argue that this is not always a reliable criterion, as the binding strengths for the same protein-ligand complex can vary between different experiments due to differences in experimental conditions, measurement techniques, experimental errors, and other non-structural factors. You report finding 19 pairs of complexes that did not pass the filter. Could you please provide the raw computed results for pairwise protein similarity, ligand similarity, and binding strengths?

Response: Thanks for this comment. We agree that including the criterion of '*different binding strengths*' may not be a reliable strategy, and therefore we use the two-dimensional similarity test (protein similarity plus ligand similarity) to clean the data. The similarity statistics after the cleaning is shown in the figure above. Moreover, the updated PDB lists for Training, Validation, Test1 and Test2 sets are uploaded to our *Zenodo* repository.

Specifically, we adopted *R* for the similarity tests. To acquire the similarities between pairwise protein sequences, the *crossSetSim* function from the *protr* library was employed, with the default substitution matrix of BLOSUM62. The *cmp.similarity* function from the *ChemmineR* library was used for calculating the pairwise ligand similarities based on the SMILES-transformed descriptors. The similarity values are presented in the above figure (*Figure 4 of the revised manuscript*).
