# Supplementary material for: Scoring protein-ligand binding structures through learning atomic graphs with inter-molecular adjacency
Source: PLoS Comput Biol. 2025 May 9;21(5):e1013074. doi: 10.1371/journal.pcbi.1013074 (PMC12091896; doi:10.1371/journal.pcbi.1013074)
Supplement: Attachment — Submitted filename: ResponseLetter_auresp_4.pdf [file pcbi.1013074.s009.pdf]

Response Letter

We would like to thank the editor and reviewer again for their helpful comments. We have revised the manuscript accordingly and provided the responses as follows.

Reviewer 2

1. In the new version of their work, the authors have taken steps to ensure that the training and test sets contain sufficiently distinct proteins and ligands. I believe these measures provide a more robust and reliable evaluation of the model. However, I have identified additional concerns regarding the validation and test sets:

The validation set and test1 set share the following complexes: 1vso, 2p4y, 2pog, 2qbq, 2qbr, 2vkm, 2vw5, 3bgz, 3e92, 3f3d.
The validation set and test2 set share the following complexes: 1gpk, 1h22, 1h23, 1nc1, 1nc3, 1ps3, 1qkt, 1r5y, 1s38, 1yc1, 1z95, 2fvd, 2hb1.

Keeping the validation and test sets completely disjoint is the standard best practice to ensure a robust and unbiased model performance estimate. Additionally, I believe the same protocol used to ensure dissimilarity between the training and test sets should be applied to guarantee that no similar proteins or ligands are shared:

- Between the validation set and the test sets
- Among the test sets themselves

Again, this will help maintain consistency and prevent potential data leakage, ensuring a more robust and reliable evaluation of the model.

Response: Thanks for this comment that leads to a more robust evaluation of the models. In the previous version, we focused on avoiding data leakage between the training set and the validation/test sets, but overlooked the check of validation and test sets themselves. Based on the previous protocol (protein sequence similarity < 0.3 or ligand similarity < 0.7), we have further checked the similarity between the validation set and each test set, and that between the two test sets (Test1 and Test2).

1) Step 1: Validation set vs. Test1 set. 10 complexes (IDs of *1vso, 2p4y, 2pog, 2qbq, 2qbr, 2vkm, 2vw5, 3bgz, 3e92* and *3f3d*) were removed from Test1 set.
2) Step 2: Validation set vs. Test2 set. 13 complexes (IDs of *1gpk, 1h22, 1h23, 1nc1, 1nc3, 1ps3, 1qkt, 1r5y, 1s38, 1yc1, 1z95, 2fvd* and *2hb1*) were removed from Test2 set.
3) Step 3: Test1 set vs. Test2 set. 3 complexes (IDs of *1gzc, 1toj* and *1ax2*) were removed from Test2 set.

Accordingly, there are 116 and 102 complexes left in Test1 and Test2 sets, which can be found in the *Zenodo* repository (https://doi.org/10.5281/zenodo.14862107). Since the training data (5007 complexes) and validation data (195 complexes) are not affected,

the previously trained models are still valid. ***Scoring performance evaluation.*** We have applied the trained models to the prediction of binding affinities for the complexes in the updated test sets. Such new results have been provided in the revised manuscript (Table 2, Page 10). According to these new results, the model performances do not vary much and the AGIMA models are still competitive. More details can be found in the section of *Experiment and Results – Scoring Performance of Models* (Lines 206 ~ 213 and 216 ~ 235, Page 9). ***Following analysis.*** As the following analysis was mainly based on the trained models (not affected), the earlier analysis results are still valid. Overall, the proposed AGIMA models are a good performer in scoring tasks.

2. In addition, I have the following suggestions and comments:

(1) It would be very useful for the scientific community to have access to a Docker or Singularity container, or a server, with trained AGIMA models available for use. This would facilitate reproducibility and allow other researchers to test and build upon your work without requiring training the model from scratch, etc.

Response: Thanks for this comment. We regret that we are currently unable to provide public access to a server. Instead, *we have saved the trained model (AGIMA-Score[18]) in Keras format and uploaded it to the Zenodo repository to facilitate the public usage*. In the future, we will try to consolidate the data, tools, and documents into a web application that will accept prediction queries.

(2) To further ensure reproducibility, please include the model's predictions in the Zenodo repository together with the rest of the data.

Response: Thanks for this suggestion. We have now provided the predictions generated by the *AGIMA-Score[18]* model for the Validation, Test1 and Test2 sets.

(3) Please provide a description of the protocol used to ensure dissimilarity between proteins and ligands in the training and test datasets (software used, thresholds applied, etc). The method is described in the author's answer to the reviewers but has not been included in the paper.

Response: Thanks for this suggestion. The description of the similarity-test protocol has now been included in the revised manuscript. More details can be found in the section of *Experiment and Results – Scoring Performance of Models* (Lines 196 ~ 203, Page 8).

3. I have noticed several typographical issues that should be addressed. A thorough proofread and a minor revision of the text would improve clarity and readability. For example:

(1) Line 209 (Person's -> Pearson's)

(2) Line 66 ("However, designing task-oriented graph edges, particularly for those molecule-involved tasks, is always a challenging task.")

Response: Thanks for this suggestion. We have corrected these typos and proofread the manuscript to improve readability further.
