# Supplementary material for: Scoring protein-ligand binding structures through learning atomic graphs with inter-molecular adjacency
Source: PLoS Comput Biol. 2025 May 9;21(5):e1013074. doi: 10.1371/journal.pcbi.1013074 (PMC12091896; doi:10.1371/journal.pcbi.1013074)
Supplement: Attachment — Submitted filename: ResponseLetter_auresp_5.pdf [file pcbi.1013074.s010.pdf]

Response Letter

We would like to thank the editor and reviewer again for their helpful comments. We have revised the manuscript accordingly and provided the responses as follows.

Reviewer 2

1. It appears that the files test1.zip and test2.zip uploaded to the Zenodo repository contain identical data. Please verify and correct this to ensure each test set is accurately provided.

Response: Sorry for this careless mistake. We have updated test2.zip in the new version of the repository in *Zenodo* (https://zenodo.org/records/15023336).

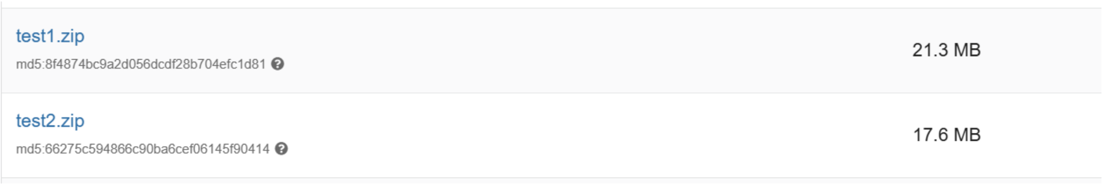

Fig 1. Updated record for Test1 and Test2 data in *Zenodo*.

2. While I appreciate that the authors have uploaded the model as a Keras file to Zenodo, I believe this alone is insufficient for optimal reproducibility and ease of use by the scientific community. If building a web server is not possible, I strongly suggest providing a Docker or Singularity container with the trained AGIMA model pre-installed, along with a clear use-case example demonstrating its application. This approach would greatly facilitate reproducibility and allow other researchers to test and build upon your work without requiring extensive setup or re-training of the model.

Response: Thanks for this suggestion. We have added two Docker containers to the *Zenodo* repository, designed for two different application scenarios. They can be run on a Unix/Linux system. The 'NOTE.txt' shows how to use these two Dockers.

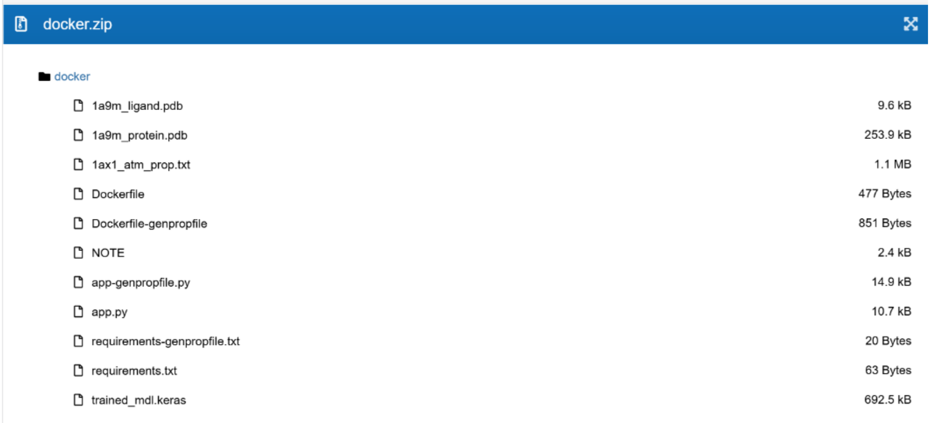

Fig 2. Two Docker containers are added to the *Zenodo* repository.

(1) Scenario 1: predicting the binding affinity for a protein-ligand complex with the ***atom property file of the complex*** as an input. This Docker container (‘*Docerfile*’) has the trained model ‘*trained_mdl.keras*’ pre-installed (here ‘*requirements.txt*’ is the list of required packages and ‘*app.py*’ is the Python file for running the model). We can build and run this Docker as follows:

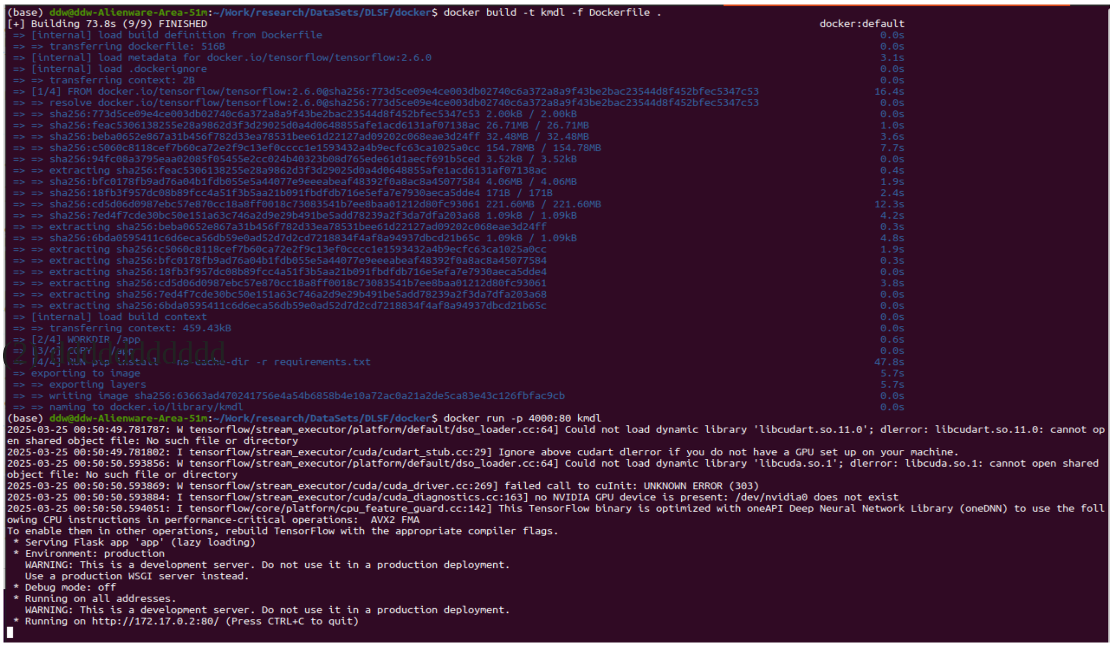

Then you can test this API by providing an atom property file (e.g. ‘1ax1_atm_prop.txt’ in *Zenodo* or your own file). The output 4.032 indicates the predicted binding affinity, as shown below.

```
(base) ddw@ddw-Alienware-Area-51m:~/Work/research/DataSets/DLSF/docker$ curl -X POST -H "Content-Type: application/json" -d '{"file": "1ax1_atm_prop.txt"}' http://localhost:4000/json
{"prediction":4.032131671905518}
```

(2) Scenario 2: predicting the binding affinity for a protein-ligand complex with the ***protein PDB file and ligand PDB file*** as inputs (same as in the original *PDBbind* database). We can first generate the atom property file of the complex using another Docker container (‘*Docerfile-genpropfile*’), with the list of required packages in ‘*requirements-genpropfile.txt*’ and the Python code in ‘*app-genpropfile.py*’. Build and run this Docker as follows:

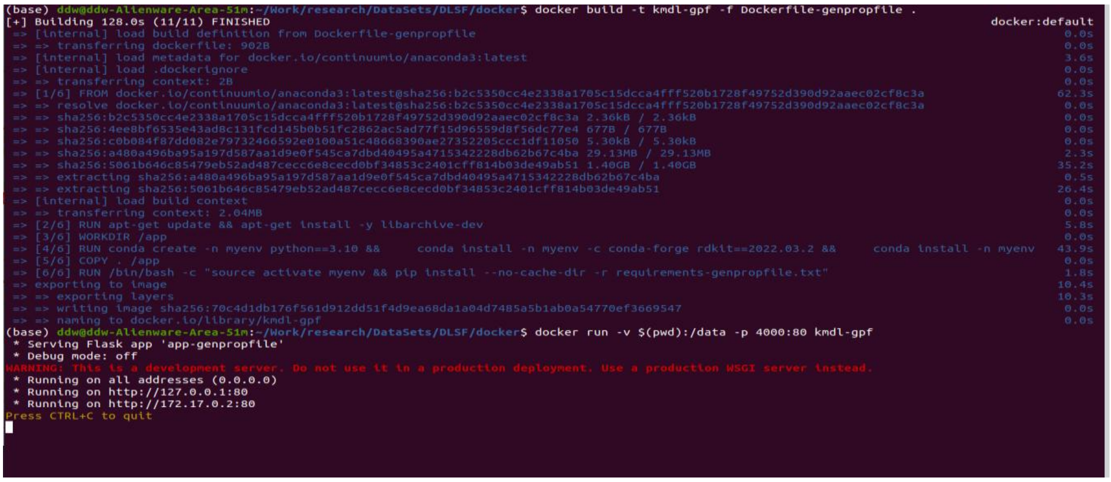

Then we generate the atom property file by providing the protein PDB file and ligand PDB file for a complex (e.g. ‘1a9m_protein.pdb’ and ‘1a9m_ligand.pdb’ in *Zenodo* or your own files).

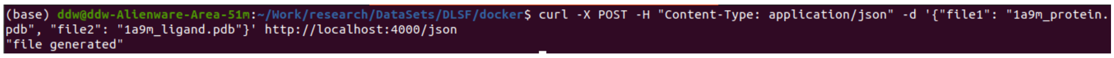

After this step, an atom property file will be generated for this complex in the docker folder, as shown below:

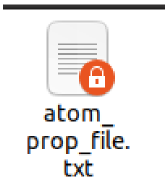

Then you can close the above Docker and run the Docker in Scenario 1 to predict the binding affinity using this atom property file.

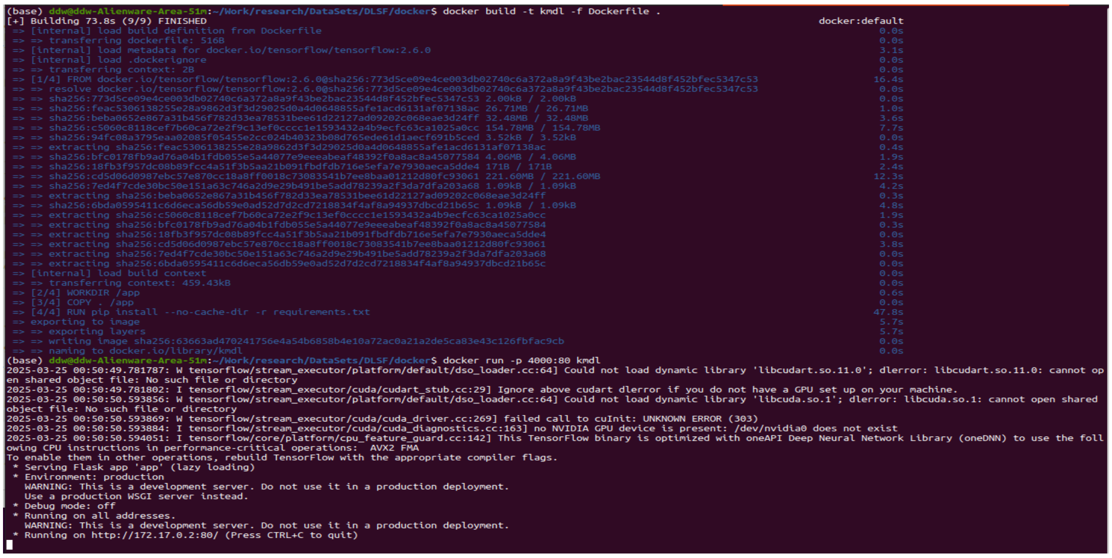

Predict the binding affinity using the API as follows. The output 6.769 shows the predicted binding affinity of the corresponding complex.

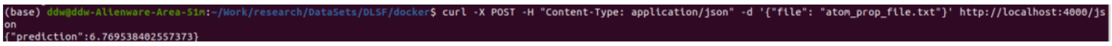

3. I suggest to explicitly indicate in the manuscript that the trained model is available in the Zenodo repository, clearly specifying the format and instructions for usage.

Response: Thanks for this suggestion. We have indicated in the revised manuscript that a Docker container with the trained model pre-installed is available in the *Zenodo* repository (mentioned in both *Abstract* and the Section of *Experiment and Results - Scoring Performance of Models*). Due to the length limitation of the manuscript file, the descriptions for the Dockers have been added on *Zenodo* and the instructions for usage have been provided in ‘NOTE.txt’ on *Zenodo* (with files for demonstration: 1ax1_atm_prop.txt’, ‘1a9m_protein.pdb’ and ‘1a9m_ligand.pdb’).
